# Understanding Barriers to Novel Data Linkages: Topic Modeling of the Results of the LifeInfo Survey

**DOI:** 10.2196/24236

**Published:** 2021-05-17

**Authors:** Holly Clarke, Stephen Clark, Mark Birkin, Heather Iles-Smith, Adam Glaser, Michelle A Morris

**Affiliations:** 1 Leeds Institute for Data Analytics University of Leeds Leeds United Kingdom; 2 School of Geography University of Leeds Leeds United Kingdom; 3 Leeds Teaching Hospitals NHS Trust Leeds United Kingdom; 4 School of Health and Society University of Salford Salford United Kingdom; 5 Leeds Institute of Medical Research School of Medicine University of Leeds Leeds United Kingdom

**Keywords:** topic modeling, text analysis, lifestyle data, consumer data, mHealth, loyalty card, fitness tracker, data linkage, data sharing, public attitudes, public opinion

## Abstract

**Background:**

Novel consumer and lifestyle data, such as those collected by supermarket loyalty cards or mobile phone exercise tracking apps, offer numerous benefits for researchers seeking to understand diet- and exercise-related risk factors for diseases. However, limited research has addressed public attitudes toward linking these data with individual health records for research purposes. Data linkage, combining data from multiple sources, provides the opportunity to enhance preexisting data sets to gain new insights.

**Objective:**

The aim of this study is to identify key barriers to data linkage and recommend safeguards and procedures that would encourage individuals to share such data for potential future research.

**Methods:**

The *LifeInfo Survey* consulted the public on their attitudes toward sharing consumer and lifestyle data for research purposes. Where barriers to data sharing existed, participants provided unstructured survey responses detailing what would make them more likely to share data for linkage with their health records in the future. The topic modeling technique latent Dirichlet allocation was used to analyze these textual responses to uncover common thematic topics within the texts.

**Results:**

Participants provided responses related to sharing their store loyalty card data (n=2338) and health and fitness app data (n=1531). Key barriers to data sharing identified through topic modeling included data safety and security, personal privacy, requirements of further information, fear of data being accessed by others, problems with data accuracy, not understanding the reason for data linkage, and not using services that produce these data. We provide recommendations for addressing these issues to establish the best practice for future researchers interested in using these data.

**Conclusions:**

This study formulates a large-scale consultation of public attitudes toward this kind of data linkage, which is an important first step in understanding and addressing barriers to participation in research using novel consumer and lifestyle data.

## Introduction

### Background

Poor diet and physical inactivity are known to contribute to millions of early deaths worldwide [1,2]. In the United Kingdom, 1 in 7 deaths are attributed to poor diet, whereas 1 in 6 deaths are attributed to physical inactivity [3,4]. A greater understanding of these risk factors for lifestyle-influenced diseases such as type 2 diabetes, certain cancers, and cardiovascular diseases is needed to improve global health. At the same time, technological advancements have led to increasingly large volumes of *big data* being produced about individual food consumption and exercise habits [5,6].

Historically, a major barrier to research on lifestyle risk factors for noncommunicable diseases has been the availability of accurate, robust, and reproducible data on diet and exercise [7,8]. Big and novel lifestyle data, produced when using services such as supermarket loyalty cards or health and fitness monitoring apps, have many benefits compared with more traditional forms of data collected through surveys, interviews, and food or exercise logs; as these data are collected during everyday activities, they are naturalistic and nonintrusive [9], meaning they do not encounter the selective reporting bias entailed with traditional methods [7]. Furthermore, large volumes of data can potentially be shared with researchers almost in real time, surpassing the scale of traditional methods at a very low cost and requiring little or no effort on the part of the participant [10]. Consequently, these data are uniquely set up for at-scale longitudinal studies with the additional benefit of extending research into traditionally hard-to-reach populations [10].

These data include mobile phone step counts, GPS-tracked exercise, wearable device heart rate monitoring, and store loyalty card records. In health research, few studies have demonstrated the full utility of consumer and personal data of this sort, as they have not typically been available to researchers [7,10,11]. Nonetheless, initiatives such as the Consumer Data Research Center [12] have begun to facilitate access to novel data sources.

In the context of diet and health research, attention has particularly been drawn to the potential of using supermarket loyalty card data (eg, Tesco Club Card) to understand food and drink purchase behavior [7,10,11,13-16] and data from wearable devices (eg, Fitbit and Garmin) or mobile phone fitness apps (eg, MyFitnessPal and Strava) to understand exercise behavior [17-19]. However, studies that have used commercial lifestyle data for research purposes have reported low uptake [10]. Although this may have been influenced by factors such as the methods used to contact participants, there is a clear research need to understand participants’ reluctance to share their data.

The combination of data from multiple sources to create enhanced data sets, known as *data linkage*, provides new insights for health research that surpass those provided by the data sets individually. The value of consumer and lifestyle data is further amplified when combined with health outcomes data [10]; for example, Aiello et al [11] used supermarket loyalty card data for small geographic areas to study the association between food purchasing and health outcomes. We believe that similar work linking individual health outcomes and lifestyle data, rather than at the ecological study level, would provide added benefits through greater specificity and personalization. However, an individualized approach may highlight data privacy and ethics barriers, especially in light of understandable historical concerns regarding data linkages, such as those proposed by care.data in the United Kingdom in 2013 and 2016 [20]. In addition, data linkage can create disclosure concerns or can increase sensitivity, which must be addressed by researchers seeking to use such methods [21,22].

Public attitudes toward health data sharing for research appear to be dependent on many factors, with the most prominent being the actual data sharing process, including data security considerations [23], the purpose and social license for the research [23-25], and the level of sensitivity attributed to data [26]. Others have reported high levels of trust in health institutions, lower levels of trust in academics, and the lowest levels of trust in private companies for data sharing initiatives [24,27]. As research using big lifestyle data often involves all 3 of these factors, it is important to address how trust might influence people’s willingness to participate in research under these circumstances.

### Objectives

Despite the obvious opportunities provided by the proliferation of big data for health research, little is known about public attitudes toward the linkage of lifestyle data with individual health records for research. The *LifeInfo Survey* is the first of its kind at this scale (n=7101 participants) to consult the public on their attitudes toward sharing novel forms of consumer and lifestyle data for linkage with their health records for health research [28]. Moreover, this survey included free-text response questions that allowed individuals, in their own words, to state what actions would alleviate their concerns about data sharing in this setting. Surveys do not frequently allow for unstructured answers of this kind because of the subjectivity and time commitment imposed with the qualitative coding of texts [29]. The use of novel data science methods, including topic modeling, can facilitate the semiautomated analysis of large amounts of textual data to identify latent themes [30].

This study aims to advance the understanding of attitudes toward data sharing by identifying specific barriers that present themselves when linking store loyalty cards or health and fitness app data with individual health records for research purposes. We recommend procedures and safeguards that can be applied to future research linking lifestyle and health data to increase participant support.

We hypothesize that common issues identified in the literature on public attitudes toward data sharing for health research, such as data security and trust, will be evident within survey responses in addition to concerns specific to the type of data to be linked, in this case, store loyalty card and health and fitness app data.

## Methods

### Data Collection

To gather public opinion on data sharing and linkage, the *LifeInfo Survey* recruited participants between September 2017 and October 2019 across 2 health settings, the Leeds Teaching Hospitals National Health Service Trust and Low Moor Medical General Practice Surgery, and 2 nonhealth settings, the Leeds Institute for Data Analytics and the Leeds City Council Health Communities survey [31]. The *LifeInfo Survey* (Multimedia Appendix 1) addressed a hypothetical scenario—whether, if asked for a future study, respondents would give permission for their consumer and lifestyle data to be linked with their health records for health research. This was conditional on their data being stored safely and not shared with anyone outside the research team. The survey consulted participants specifically about 2 types of data: (1) consumer data from store loyalty cards detailing food and drink purchases and (2) lifestyle data from health and fitness apps, websites, and wearable devices. Basic demographic data and which specific loyalty cards and health and fitness apps respondents used were additionally captured. Additional information about the *LifeInfo* project can be found in the study protocol [32]. Detailed information on the participants and the main survey results are reported elsewhere [28].

Those who responded *no* or *not sure* to whether they would share their data were asked, “what (if anything) might make you change your mind in the future?” concerning their (1) store loyalty card data and (2) health and fitness app data. This study primarily analyzes the qualitative responses to these 2 questions (questions 4 and 9 in the original questionnaire included in Multimedia Appendix 1), henceforth referred to as (1) *the store loyalty card question* and (2) *the health and fitness app question*, to answer the following research question: “What are the reported barriers to linkage of lifestyle data with health records for research?” Responses were given in free-text format, allowing individuals to state, in their own words, potential desired changes that would make them more willing to share such data, although many also used this space to explain their reasons behind more negative responses. Primary analysis regarding overall willingness to share lifestyle and data demographic trends are reported elsewhere [28] and summarized below. The survey questionnaire and all responses were provided in English.

### Ethics

This study was granted ethical approval by the London-Brent Research Ethics Committee (reference 17/LO/0622).

### Modeling

Latent Dirichlet allocation (LDA) was applied as a method of automated content analysis on unstructured survey responses. This technique was used to identify the underlying factors that contribute to respondents’ unwillingness or unsureness to having their consumer or lifestyle data and health records linked for research purposes and potential changes that could influence them to do so. LDA is a generative probabilistic model that is frequently applied to textual data. The model has a 3-level hierarchical Bayesian structure under which each *document* is modeled as several topics, and each topic is modeled as a set of terms [30]. The model uses the Gibbs sampling technique to estimate model parameters. The LDA modeling procedure was applied to free-text responses separately for the store loyalty card question and the health and fitness app question to create a model for each.

#### Processing

Data cleaning and processing were performed using the R software [33]. Noninformative responses (eg, *N/A* and *no comment*) were removed from the data set. Survey responses that consisted of only a single word were removed from the analysis data set, as the underlying mechanisms of LDA are based on the co-occurrence of terms. These are categorized separately, as attitudes are easily ascertained from single-word responses (shown in Tables 1 and 2).

**Table 1:** Counts of single-word responses to the question, “What (if anything) might make you change your mind in the future?” about sharing store loyalty card data for linkage with health records (n=396).

Single word	Counts per word, n (%)
Nothing	256 (64.6)
No	98 (24.8)
Privacy	10 (2.5)
None	8 (2.0)
Maybe	5 (1.2)
Private	3 (0.8)
Confidentiality; personal	2 (0.5)
Confidential; discounts; dk^a^; hackers; illegible; incentives; money; unneeded; unlikely; unsure; why; yes	1 (0.3)

^a^dk: don’t know.

**Table 2:** Counts of single-word responses to the question, “What (if anything) might make you change your mind in the future?” about sharing fitness app data for linkage with health records (n=309).

Single word	Count per word, n (%)
Nothing	189 (61.2)
No	71 (23.0)
Privacy; security	7 (2.3)
Maybe; none	5 (1.6)
q4^a^	4 (1.3)
Anonymity; confidential; private; same; yes	2 (0.6)
Benefit; confidentiality; intrusive; might; nil; personal; possibly; relevance; uncertain; unlikely; why	1 (0.3)

^a^q4: question 4.

Preprocessing procedures, standard in natural language processing, were undertaken to create a *document term matrix* (DTM) on which to perform LDA. This included converting all words to lower case and removing white spaces, punctuations, and common *stop-words* from texts to leave only meaningful words. Frequent misspellings were replaced with their correct form, and common equivalent words were standardized (Multimedia Appendix 2).

The *SMART* (System for the Mechanical Analysis and Retrieval of Text) stop-word data set was used to identify uninformative words. However, bigrams (two-word terms) that contain many common stop-words were not removed to uncover attitude positions within responses (Multimedia Appendix 2). Numbers were not removed as numeric bigrams are potentially meaningful (eg, *100 percent* and *3rd party*). Finally, lemmatization of words was undertaken to convert words into their root form (*lemma*) and reduce sparsity within the DTM (eg, *cards* was replaced with *card*; Multimedia Appendix 2). Lemmatization was preferred over stemming, as lemmas are more human-readable than stems, which are not always complete words (eg, *storing* would be replaced with the stem *stor*).

LDA was performed on a DTM of unigrams (one-word terms) and bigrams (two-word terms) within the texts. Bigrams are included in the DTM as this creates more human-interpretable topics, and many words are context-specific, for example, *big brother*/*change mind*, or formulate attitude positions in combination, for example, *would change*/*wouldn’t change*. In the example given, the term *brother* alone would provide little insight into data sharing attitudes, yet *big brother* signifies a potential invasion of privacy and distrust. Several papers have reported improved results for including bigrams and higher n-grams in different topic models [34-36]. Only terms that occurred in more than 2 documents were retained, leaving 993 unique terms for the store loyalty card question and 554 unique terms for the health and fitness app question.

#### Topic Number Selection

Selecting an appropriate number of topics is a key challenge for LDA. According to Green et al [37], “too few topics will produce results that are overly broad, whereas choosing too many will result in the ‘over-clustering’ of a corpus into many small, highly-similar topics” that are difficult to interpret in a meaningful way. The number of topics (k) is conventionally chosen as the model with the lowest value of *perplexity* when applying different models of candidate k to held-out data [30,38]. This perplexity measure captures how well a probability distribution or probability model predicts a sample, indicating how *surprised* the model is by new data. For LDA, it is equivalent to the inverse of the geometric mean per-word likelihood calculated on the held-out data [30]. For this analysis, ten-fold crossvalidation was used to select an appropriate number of topics (k) from a candidate list of 15 k ranging from 2 to 100, optimizing for perplexity. Candidate k increases in smaller intervals between lower values, as greater change is expected between these values. The crossvalidation process randomly divides the data set into 10 approximately equally sized folds and uses 9 of these to train the model, using the held-out fold to test the model. This process was repeated 10 times such that each fold was used as the testing set once.

Some research has found that the models that produce the most semantically meaningful topics—in that topics are easily interpreted by humans and terms representing concepts are given high probabilities within the model—are not necessarily the models with the best perplexity scores [39]. Hence, a measurement of *coherence*, which research finds corresponds well with human-interpretable topics [40], was also considered. Average topic *probabilistic coherence* measures topic quality based on how commonly topic terms co-occur, controlling for statistical independence [41-43]. This was compared for models of candidate k topics and balanced with perplexity scores.

Once an appropriate number of topics were selected for each LDA model, the final models were created using responses from all individuals for the given question. This method was chosen, rather than using commonly used training and testing approaches, to support the study aim of summarizing survey responses rather than creating a predictive model to categorize new data, as no new *LifeInfo Survey* data will be collected in the future. Moreover, the size of the data set is small compared with many others that use LDA, and splitting the data set into fewer responses could reduce the model quality.

For both topic number selection and the final models, the LDA hyperparameters were set at α=.1, influencing document-topic density, and β=.05, influencing word-topic density. The α prior was set at this relatively low value because *LifeInfo Survey* responses were short (refer to Multimedia Appendix 3 for plots showing the distribution of response lengths in words, averaging 12 words per document for the store loyalty card question and 9 words for the health and fitness app question), so we would expect there to be only a few topics formulating each document. β was also set to a relatively low value, as we expected a small number of words to be highly influential per topic given the short responses. α is modeled as asymmetrical, as we expected some topics to be more common than others within the survey responses. Previous work has found that asymmetrical α values provide substantial advantages to LDA results, whereas asymmetrical β priors provide no benefit [44].

#### Hierarchical Clustering

To make the topics more easily interpretable, those created through LDA modeling were further categorized into thematic groups with the aid of hierarchical clustering of topics (Multimedia Appendix 4). These topics were given summarizing names, shown in Tables 3 and 4 by subheadings in bold, based on their content, considering both topic *top terms* and the contextual use of these terms in texts (Multimedia Appendix 5). The Hellinger distance [45] between topics was calculated based on their term ϕ values, and the 2 closest topics were clustered together. ϕ values are the probability of a term being used within a text given; therefore, topics that more frequently use the same terms are considered closer. This was done iteratively until hierarchical clusters split topics into meaningful thematic categories. In some cases, topics thematically aligned with a category that was found to be semantically different to the topic according to hierarchical clustering; these are marked with a superscript in the tables and dendrogram and were reassigned to their appropriate thematic category.

**Table 3:** Topics created by latent Dirichlet allocation modeling of LifeInfo store loyalty card question, showing topic names, top 15 terms per topic according to term ϕ values, topic prevalence, and topic probabilistic coherence. In total, 9 thematic categories are shown.

Themes and SC^a^ topic number		Topic name: top 15 terms selected by highest probability of the term given the topic	Prevalence (estimated survey responses; n=1930)^b^, n (%)	Coherence
**Nothing would change mind**				
	SC14	Wouldn’t change mind: change, mind, change mind, nothing, would change, nothing would, don’t think, make, wouldn’t change, nothing change, think would, anything would, make change, think anything, and would make	134.52 (6.97)	0.47
**Store loyalty card/don’t use store card**				
	SC16	Store loyalty card/don’t use: card, store, loyalty, loyalty card, store card, health, store loyalty, link, card health, don’t use, use loyalty, why store, no need, card would, and use store	147.65 (7.65)	0.34
“**Big Brother” and privacy invasion**				
	SC8	Big brother/nanny state: feel, privacy, big brother, brother, nothing, invasion, invasion privacy, regard, don’t feel, thing, feel like, state, choice, watch, and nanny	80.10 (4.15)	0.16
	SC1	Privacy and cold calling: concern, privacy, concern about, confidentiality, would concern, email, require, call, future, bombard, advertise, market, guarantee, about privacy, and would require	73.15 (3.79)	0.13
**Personal information sharing and access by others**				
	SC2	Concerns about linkage and insurance: information, give, idea, insurance, don’t like, health, like idea, good, information could, wrong, reassurance, health insurance, hand, affect, and know about	80.87 (4.19)	0.05
	SC19	Data access and others: information, private, personal, access, company, stored, detail, hold, information stored, sell, people, safe, personal information, personal health, and party	103.45 (5.36)	0.04
	SC3	Don’t want to share personal information: share, information, information share, personal, share information, personal information, don’t want, nothing, detail, want information, nothing don’t, want share, wouldn’t want, don’t like, and not share	100.36 (5.20)	0.12
**Data inaccuracy**				
	SC7	Data inaccuracy and bias: buy, shop, make, purchase, family, people, food, eat, lifestyle, relate, supermarket, diet, healthy, product, and good	117.92 (6.11)	0.09
**Data security and protection**				
	SC15	Don’t trust organizations with data: data, trust, don’t trust, NHS^c^, share, organization, system, personal data, data share, hack, personal, guarantee, secure, not trust, and safety	95.34 (4.94)	0.09
	SC12	Data protection: data, data protection, protection, data would, data use, access, issue, how data, health data, would use, health, wouldn’t want, data link, secure, and link	81.06 (4.20)	0.14
	SC9	Data security: security, data, data security, breach, worry, assurance, worry about, security data, data breach, about security, information security, increase, improve, risk, and security information	82.41 (4.27)	0.06
	SC5	Guaranteed data safety/security: secure, 100, 100 percent, percent, convince, stored, safely, safe, stored safely, prefer, separate, if could, NHS, control, and how secure	74.88 (3.88)	0.37
**Understanding research purpose and process**				
	SC20	Don’t understand benefit: understand, benefit, don’t understand, why would, would need, understand why, necessary, link, purpose, need understand, understand benefit, understand purpose, why need, would necessary, and need link	82.41 (4.27)	0.17
	SC6	Require demonstratable benefits: benefit, not sure, benefit would, link, explanation, see benefit, explain, appropriate, explanation why, would benefit, explain benefit, if could, sure would, care, and sure why	69.48 (3.60)	0.03
	SC4^d^	Require reassurance: research, depend, data, purpose, specific, would depend, study, team, anonymize, happy, access, research team, if data, contact, and not use	92.06 (4.77)	0.07
	SC18^d^	Require more information: information, more information, would use, information would, would need, would want, need know, information use, know why, want know, need more, would like, why would, information about, and how would	146.68 (7.60)	0.10
**Health records shouldn’t be linked**				
	SC17	Health record should not be linked: health, record, health record, link, link health, nothing, private, confidential, don’t know, would link, why would, health care, nothing health, record would, and care	110.20 (5.71)	0.26
	SC11^d^	Shopping habits and health shouldn’t be linked: shop, health, habit, shop habit, interest, health care, link, wouldn’t want, professional, shop health, condition, business, supermarket, health professional, and commercial	73.15 (3.79)	0.10
**Don’t understand reason/relevance of data linkage**				
	SC13	Unsure of reason for linkage: not sure, relevant, medical, would need, unsure, sure why, record, medical record, don’t see, why would, relevant health, not relevant, sure would, medical information, and sure how	92.45 (4.79)	0.04
	SC10	Don’t see reason for linkage: reason, don’t see, link, relevance, can’t see, reason why, nothing, see relevance, point, see why, 2, see reason, should link, connection, and good	91.87 (4.76)	0.08

^a^SC: store card.

^b^Total prevalence does not sum exactly to 100%, and the total survey response counts do not sum exactly to N because of rounding.

^c^NHS: National Health Service.

^d^Topic has been regrouped to the category most thematically aligned with its contents from the category hierarchical clustering indicated as semantically similar.

**Table 4:** Topics created by latent Dirichlet allocation modeling of the LifeInfo health and fitness app question.

Themes and HA^a^ topic number		Topic name: top 15 terms selected by highest probability of the term given the topic	Prevalence (estimated survey responses; n=1206)^b^, n (%)	Coherence	
**Nothing would change mind**					
	HA17	Nothing would change mind: change, mind, change mind, nothing, would change, don’t think, nothing would, think anything, make, wouldn’t change, anything would, make change, would make, future, and think would	75.86 (6.29)	0.46	
**Apps/websites/wearable devices and don’t use**					
	HA16	Device and don’t use: device, use device, not use, wear, don’t use, device not, wearable, collect, future, wearable device, data collect, wear device, app, data, and device future	59.09 (4.90)	0.10	
	HA11	App/website and don’t use: app, fitness, use app, don’t use, device, fitness app, lifestyle, app not, user, website, applicable, health app, not applicable, use fitness, and device app	53.67 (4.45)	0.13	
“**Big Brother” and privacy invasion**					
	HA3	“Big Brother:” feel, good, idea, life, big brother, brother, feel like, make, don’t like, watch, like idea, bit, exercise, control, and NHS^c^	55.8 (4.62)	0.06	
	HA2	Privacy invasion and safety/security of data: privacy, safe, store, securely, invasion, not safe, store securely, invasion privacy, feel, hacker, issue, nothing, code, data store, and partly	53.43 (4.43)	0.05	
**Personal information sharing**					
	HA20	Information would be shared: information, share, information share, information wouldn’t, store, know information, share information, wouldn’t share, information store, sure information, worry, will share, health information, would share, and information will	65.12 (5.40)	0.04	
	HA14	Information is personal: personal, information, private, access, personal information, don’t want, personal use, private information, information personal, reason, people, access information, point, long, and personal detail	61.75 (5.12)	0.08	
**Who has access to these data?**					
	HA15	Data access by insurance/private companies: company, insurance, lifestyle, relevant, make, insurance company, health, monitor, fitbit, will not, not relevant, point, interest, unsure, and wear	53.79 (4.46)	0.12	
	HA7	Health records and linkage: record, health, health record, access, link, doctor, don’t want, information, access health, food, buy, not want, put, people, and link health	63.44 (5.26)	0.20	
**Data inaccuracy**					
	HA5	Inaccurate data and partial use: accurate, phone, not accurate, step, activity, app, hold, give, run, exercise, don’t think, record, count, picture, and walk	52.58 (4.36)	0.11	
**Data security and protection**					
	HA19	Not sure and security: not sure, secure, sure how, sure would, would secure, sure about, secure would, convince, illegible, situation, how secure, sure anything, sure if, would convince, and if would	53.79 (4.46)	0.07	
	HA18	Don’t trust data security: trust, secure, nothing, don’t trust, computer, hack, fully, website, internet, not trust, nothing don’t, nothing secure, wouldn’t trust, information, and trust information	60.66 (5.03)	0.08	
	HA10^d^	Data protection against sharing: data, share, protection, data could, data protection, not share, data not, if data, share data, breach, system, sure data, thing, NHS, and bad	59.21 (4.91)	0.14	
	HA12^d^	Requires data security: security, data, concern, concern about, assurance, data security, safety, internet, information, about security, security information, use data, security would, assure, and matter	66.33 (5.50)	0.06	
**Understand research purpose and process**					
	HA6	Depends on assurances and purpose: depend, would depend, 100, 100 percent, percent, guarantee, depend how, depend information, depend use, depend why, give, depend if, depend purpose, percent guarantee, and how use	59.94 (4.97)	0.32	
	HA4	Consent to specific research: research, happy, specific, permission, study, purpose, condition, time, project, if know, only if, researcher, advance, consent, and research project	58.37 (4.84)	0.08	
	HA9^d^	Understanding how and why data would be used: benefit, data, would use, understand, would need, don’t know, data would, how would, want know, clear, would want, purpose, give, understand why, and benefit would	60 (4.98)	0.07	
	HA8^d^	Requires more information: information, would need, more information, detail, need more, need know, information would, would want, know more, why would, information use, anonymous, more about, more detail, and will use	72.84 (6.04)	0.11	
	HA1^d^	Benefits to health: health, benefit, researcher, link, professional, care, interest, health professional, health researcher, if health, health care, information, individual, would benefit, and don’t see	66.21 (5.49)	0.06	
**Same answer as question 4 (store loyalty card question)**					
	HA13	Same answer as Q4: q4, answer, 4, question, previous, question 4, answer q4, see answer, response, previous answer, see previous, answer 4, answer question, response q4, and affect	54.15 (4.49)	0.12	

^a^HA: health app.

^b^Total prevalence does not sum exactly to 100%, and the total survey response counts do not sum exactly to N because of rounding.

^c^NHS: National Health Service.

^d^Topic has been regrouped to the category most thematically aligned with its contents from the category hierarchical clustering indicated as semantically similar.

#### Making Recommendations

The recommended actions that researchers can take to address the key barriers to data sharing and linkage identified through LDA modeling are presented. These recommendations are based on the synthesis of participant suggestions and expertise regarding wider research on data sharing.

## Results

### Data Collection

The *LifeInfo Survey* recruited 7101 participants. The primary results of this study are reported elsewhere [28]. In brief, of those who reported using the services, 51.50% (2521/4895) responded favorably to sharing their loyalty card data for linkage to health records and 70.80% (1717/2425) responded favorably to sharing data from health and fitness apps or wearable devices to link with their health records. For the store loyalty card question, 62.28% (1489/2391) of respondents who answered *no* to whether they would share their data for linkage provided a free-text response. Of those who answered *not sure*, 66.02% (814/1233) provided a response. For the health and fitness app question, 50.82% (839/1651) of respondents who answered *no* to whether they would share their data for linkage provided a free-text response. Of those who answered *not sure*, 56.8% (565/995) provided a response.

A number of respondents who had either answered *yes* or did not provide an answer to whether they would share their data provided free-text responses that were included in the analysis (n=35 for the store loyalty card question and n=127 for the health and fitness app question). In total, 2338 individuals provided a free-text response to the store loyalty card question and 1531 for the health and fitness app question. Preprocessing steps and removing single-word responses reduced the number of responses to 1930 for the store loyalty card question and 1206 for the health and fitness app question. Single-word responses were considered separately and are shown in Tables 1 and 2.

### Health and Fitness Modeling

#### Topic Number Selection

**Figure 1:**
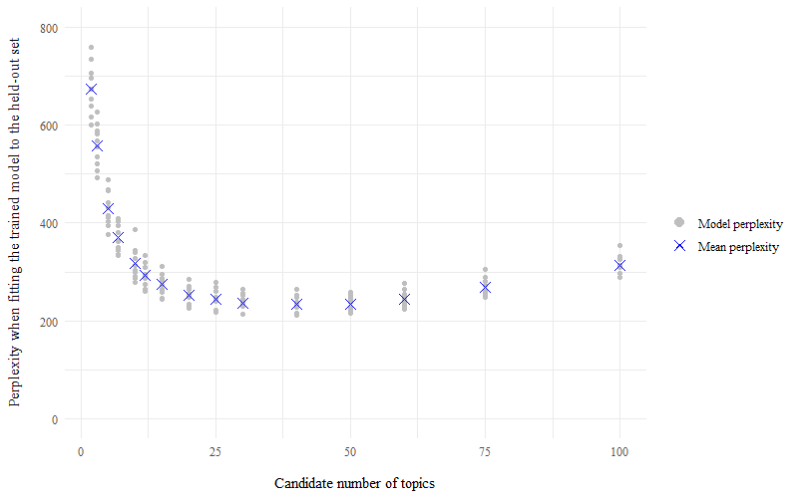
Perplexity scores for the models—LifeInfo store loyalty card question, 10-fold cross-validation of topic modelling to establish the optimal number of topics for latent Dirichlet allocation.

**Figure 2:**
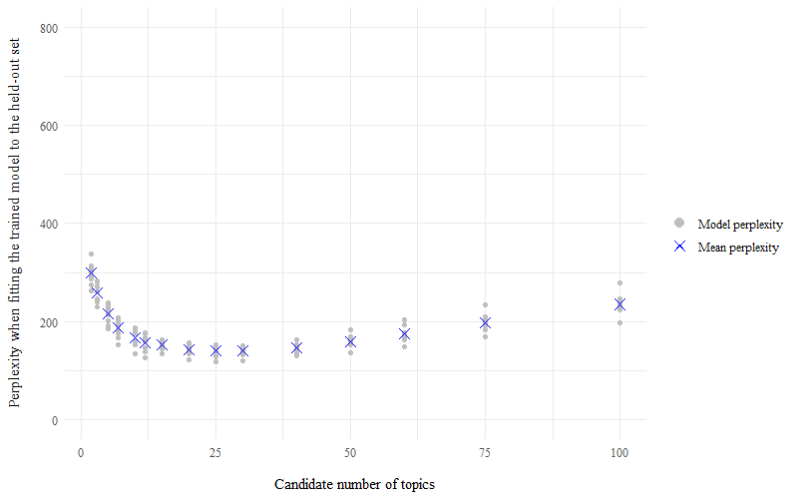
Perplexity scores for the models—LifeInfo health and fitness app question, 10-fold cross-validation of topic modelling to establish the optimal number of topics for latent Dirichlet allocation.

**Figure 3:**
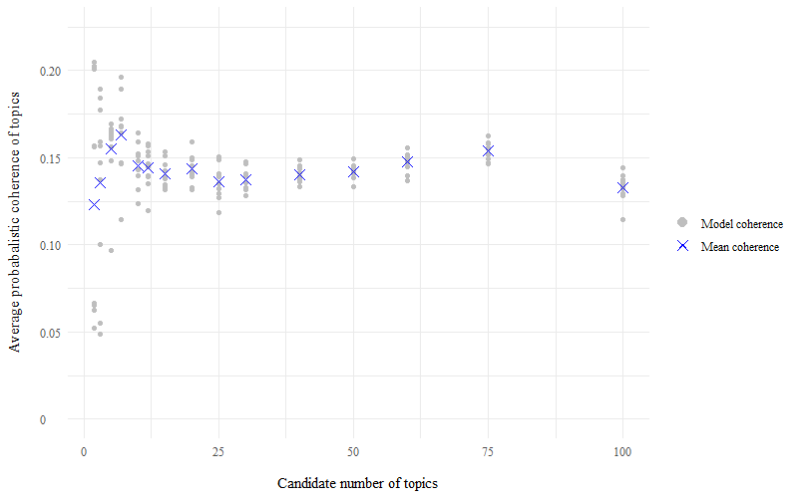
Average probabilistic coherence scores for the models—LifeInfo store loyalty card question, 10-fold cross-validation of topic modelling to establish the optimal number of topics for latent Dirichlet allocation.

**Figure 4:**
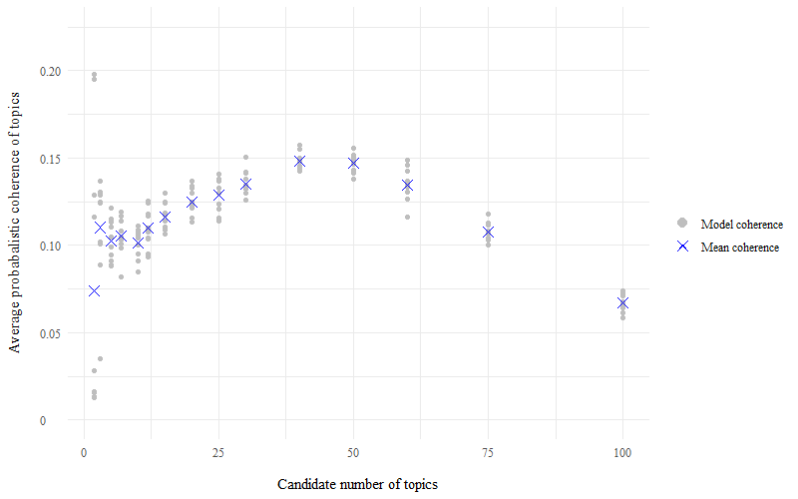
Average probabilistic coherence scores for the models—LifeInfo health and fitness app question, 10-fold cross-validation of topic modelling to establish the optimal number of topics for latent Dirichlet allocation.

For the store loyalty card question, perplexity scores for each model of candidate number of topics (k) indicated that the best model has a number of topics within the range of k=20 to k=60, as the perplexity scores plateau at their minimum value within this range (Figure 1). For the health and fitness app question, perplexity scores were minimized to within the topic number range of 20-30 topics (Figure 2). The average probabilistic coherence scores within these ranges of k varied by only very small amounts, indicating that the models had near-equivalent topic quality (Figures 3 and 4). To maximize human interpretation and for comparability between the 2 questions, parsimonious models of 20 topics were chosen for both the store loyalty card question and health and fitness app question.

#### LDA Topics

Table 3 shows the 20 topics created by the final LDA model of free-text responses to the store loyalty card question. The table includes the 15 top terms most representative of each topic, selected according to their ϕ values. As ϕ values are the probability of a term being used within a text given the topic, those terms with the highest ϕ values are representative of the topic content. The table includes 2 further topic measures: first, topic prevalence, which indicates how common each topic was within the survey responses (eg, prevalence 5.0 indicates 5% of survey responses fell into this topic), and second, probabilistic coherence, as an indicator of the semantic meaningfulness of each topic (this can range from 0 to 1, where higher numbers are more meaningful). Lower values of probabilistic coherence are owing to lower frequencies of topic term co-occurrences within texts and/or topic terms being highly frequent within the data set at large. Low coherence scores are associated with conceptually less defined topics [40]; however, they could also be caused by *fuzzy* topics with many different terms or semantic crossover within other topics. Table 4 shows the same information for the 20 topics created by LDA modeling of the health and fitness app question responses.

LDA modeling also assigns survey responses with probabilities for each topic, known as *θ values* A higher θ value for any given topic indicates that the response should more likely be categorized into that topic (Multimedia Appendix 5 shows example responses most associated with each topic). Some responses, especially those that are very short (consisting of only 1 substantive term), are given equal θ values across all topics, which can be regarded as *uncategorizable* (Multimedia Appendix 6). There are some differences in LDA topic frequency depending on demographic groups (eg, age and gender); further analysis demonstrating demographic patterns can be found in Multimedia Appendix 7. Topic stability is validated by comparing results across multiple LDA runs (Multimedia Appendix 8), indicating that the thematic categories reported are consistently produced in topics created through LDA.

## Discussion

### Principal Findings

Understanding attitudes toward using big lifestyle data for health research is important for the success of research initiatives interested in using these data in the future. More than half of our participants who generated big lifestyle data reported that they would be happy for these data to be linked to health records for future research. As only individuals who stated that they were unwilling or unsure about sharing their lifestyle data were prompted to respond to what would make them change their mind, these topics identified specific barriers to data sharing. Topic modeling on survey responses produced thematic topics that summarized latent themes of concern to potential data subjects. We believe that the intelligence generated will support researchers in addressing these issues in the future with the appropriate use of safeguards and consent procedures to generate a publicly acceptable study design.

It is also worth noting that, although LDA modeling aims to create distinct topics, individual responses may discuss multiple topics, and many of the topics identified were interconnected and complementary. In addition, topic quality varies and can be inferred by topic probabilistic coherence scores, which indicate how clearly defined each topic is semantically. For example, topic HA2 (Table 4) had a low value of probabilistic coherence (0.05) and primarily focused on the issue of privacy invasion; however, it also discussed the safety and security of data.

### Barriers to Data Linkage

The topics uncovered by LDA modeling indicated that many of the same issues arise for both sharing store loyalty cards and health and fitness app data. Many topics can be matched across Tables 3 and 4 or highlight similar themes. Overall, key barriers to the use and linkage of store loyalty cards and physical activity data for health research included data safety and security, personal privacy, the need for further understanding about the research and study purpose, fear that data could get into the *wrong hands*, problems with data accuracy, and not understanding the reason for data linkage. These barriers can potentially be addressed by researchers with varying degrees of ease. However, for some respondents, nothing would make them share these data, whereas others did not use store loyalty cards or health and fitness apps. Many of these issues are common in the literature on health, consumer, and personal data sharing, and as such, these are expected findings; however, new concerns also arise specific to individual data linkages. Example responses most associated with each topic are used throughout this discussion and are labeled with their relevant topic. These responses were selected as those with the highest probabilities of being categorized into a given topic and can be viewed in Multimedia Appendix 5.

#### Nothing Would Change My Mind

Among those who responded negatively to sharing their lifestyle data for health research, a large proportion would be unwilling to change their mind. This is indicated first by the number of texts that LDA modeling categorized as the topic *nothing would change mind*, which refers to SC14 and HA17, constituting approximately 6.94% (134/1930) and 6.30% (76/1206) of the analyzed responses, respectively, and second, by the single-word answers excluded from the analysis, which were mainly the words *nothing*, *no*, and *none* for both the store loyalty card question and the health and fitness app question (Tables 1 and 2). These 3 single-word answers combined account for 90.3% (361/396) of the single-word responses about store loyalty cards and 85.8% (265/309) of single-word responses about health and fitness apps.

#### “Don’t Use Services”

Others mentioned that they did not use these services and thus would not have data to share. This was most clearly identified within the topics related to health and fitness apps (HA16 and HA11) and was evident for store loyalty cards (SC16). For example:

I’m not going to use a wearable device in the future.

HA16

Currently don’t use store loyalty cards.

SC16

For this group, only greater participation in big lifestyle data production would allow them to share their data.

#### “Big Brother” and “Privacy Invasion”

Some respondents reported feeling that the proposed data linkage was a *big brother* and an invasion of privacy (SC8, SC1, HA2, and HA3). However, for the fitness app question, these topics were less clearly defined by LDA modeling, reflected in their low probabilistic coherence scores for these topics (0.05 and 0.06). Answers such as “not interested to a ‘big brother is watching’ on all aspects of my life” (SC8) and “[I] wouldn’t want to feel every area of my life is out of my control and being watched by an institution that already makes me feel like I have no autonomy” (HA3) indicate a dislike and feeling of *surveillance* through the data. Actions by researchers to address these feelings are limited as they are related to a broader distrust of big data; however, greater transparency as to how data are being used and assurance of anonymity may convince some users. Within the responses about store loyalty cards, SC1 specifically identified privacy concerns related to unwanted emails, phone calls, and text that can be addressed through data protection and security actions.

#### Personal Data and Linkage

Many responses focused on concerns about sharing these data, which respondents perceived as highly personal information (HA20, HA14, SC19, and SC3). References to data being personal and confidential appeared across topic categories, particularly in the context of not wanting to store loyalty card data and health records linked, data protection and security, and concerns about who is able to access these data. For example:

Nothing, this information is for my personal use. Access to my private devices can lead to security risks.

HA14

The degree to which an individual believes that their data are personal and sensitive influences their willingness to share data and with whom. Medical information is personal, particularly for individuals with complex health conditions. Health and fitness app data and store loyalty card data are personal in different ways. Fitness apps are primarily used for personal monitoring, meaning data are not created with the idea that they might be shared with other actors, whereas for store loyalty card data, individuals exchange information about purchase history with shops in exchange for discounts and points. However, in the case of transaction data, Skatova et al [13] found that people regarded the graduality of transactional data as personal.

#### “Who Has Access to Private Data?”

Health data are regarded as particularly sensitive and confidential [26], related to the theme of *private information*, and many responses mentioned worries that data would be accessed by other actors without their permission (SC2, HA15, and HA7). For example, “if the information was available only to the research team, and not to others, e.g. insurance companies, mortgage companies, even the medical team” (HA15).

Private companies, third parties, and health insurance companies were frequently mentioned by respondents as actors they feared would gain access to their data. This is expected, as research has found that these institutions are trusted least by the public to use data appropriately [24,27]. Many respondents believed these companies would use their data for profit, to increase the cost of premiums, or to deny treatment altogether. However, respondents also mentioned concerns that health care professionals would be able to access their lifestyle data. For example:

It’s my information for me. If I want my doc to know it I’ll tell him/her or put it on my health record myself.

HA7

This finding is less expected, as research finds that health care providers are one of the most trusted actors for data sharing [24,27]. This indicates that there is no straightforward relationship between an individual’s willingness to share their data and their trust in the actors involved. A further barrier for data linkage is illuminated in that data are often created for specific purposes, and alternative uses of data outside of this domain can create suspicion.

There was also a common misunderstanding that sharing lifestyle data for research would allow all involved actors to access these data. This was reflected in the previous example and others. For example, one respondent asked, “Why would I want Tesco knowing my health records?” (SC17).

Similar findings were reported by Skatova et al [13] in their research on transaction data sharing. This indicates that one easily achievable action that could influence people to share their data would be to explicitly state that data would not be available to anyone but researchers.

Closely related to data access by others was a reported general belief that health records should not be linked with other data and should be accessed only by health professionals (SC17 and SC11). This again reflects the belief that data should only be used for its designated purpose. For example, one respondent felt that “Health records should be kept in health care” (SC17).

#### Data Accuracy

Data accuracy was another identified barrier. Many respondents indicated that their purchase history or fitness tracking was only partial, creating misleading data about lifestyle behaviors (SC7 and HA5). Indeed, missingness and data integrity have been identified as a challenge for research using big lifestyle data and a concern for participants [10,26]. Nevertheless, good results have been found by comparing or combining these data with more traditional collection forms, for example, modeling individual consumption from household-level data [10,46].

When looking at responses within this topic, most participants reported concerns that their data would make them appear *less* healthy than their real activity. For example:

I buy all my fruit and veg at a farm shop...my data would provide misleading associations.

SC7

[my mobile data] shows a terrible step count, but that’s because I don’t hold my phone while playing netball, long walks etc.

HA5

Implicitly, respondents worried that they would be judged unfavorably on their lifestyle behaviors. Researchers could address these concerns by making explicit in the study protocol that (1) data are not expected to be complete, (2) detailed actions they will take to accommodate for this with modeling techniques or additional surveying, and (3) all data would be made unidentifiable foreclosing the possibility of judgment.

#### Data Protection and Security

Topics that focused on data protection and security formulated a large proportion of the responses (approximately 17.31% (334/1930) of responses for the store loyalty card question and 19.90% (240/1206) for the health and fitness app question). Changes frequently mentioned were assurances that these data would be stored completely safely and securely and that they would be protected from hacks and data breaches. These are common concerns for data sharing, especially with data that are regarded particularly private or sensitive, such as health data [26]. Although these assurances were given as a condition for data sharing within the question wording (Multimedia Appendix 1), many responses highlighted their importance or were not convinced that this would happen. Action would, therefore, need to be taken to give participants greater confidence, which could be achieved through transparency in the research process and providing details of how these data will be protected.

#### Understanding More About the Research Purpose, Process, and Benefit

Across both store loyalty cards and health and fitness app data sharing, respondents mentioned that understanding the research better and being given more control over their participation would influence them to change their mind. This includes being provided with more information (SC18 and HA8), giving permission only for specific research projects (SC4 and HA4), and a greater understanding of the reason or benefits of research (SC20, SC4, SC6, HA9, and HA1). These findings are supported by the research of Skatova et al [13], who found that support for data sharing is contingent on its context and purpose, highlighting the importance of well-informed participants. Given that the *LifeInfo Survey* aimed to assess attitudes toward data sharing in the future, rather than requesting participants to consent to data sharing at this time, the participant information sheet (Multimedia Appendix 9) needed to be suitably broad, which would not be the case when recruiting participants to an actual data linkage study.

Researchers have found that a strong social motive, such as improvements to health or treatment, in addition to clearly defining the purpose of research, are key motivating factors for personal data donation [47], whereas using health data for insurance, marketing purposes, or commercial exploitation is unacceptable to the public [23]. This was reflected in the unstructured answers from the *LifeInfo Survey*. A respondent answered that they would be supportive “if the data fed into important public health or similar research and was not used to further commercial gains by these giants of commerce” (HA4).

Again, although LDA separates topics, they are connected, and the involvement of private companies creates concerns about whether these entities will be able to access health data for profit once linked.

Control and consent for health data being used for research have been found to be key for public acceptance [25]. A respondent answered that the data “has to be linked to a condition or specific research project with additional approval provided in advance” (HA2).

This is something that can be adapted into the research process, allowing participants to consent to or deny the use of their data, given the specifications of the study.

In addition to a desire for more understanding, another thematic category was identified of individuals who did not understand the reason or relevance of data linkage (SC13 and SC10). For example, one respondent said, “Do not see any reason why they should be linked” (SC10).

These topics were only produced when modeling the store loyalty card question, although topic HA5 also encompassed responses that stated data are *not relevant*. This perhaps indicates that respondents found the link between purchasing and health to be less relevant than fitness tracking and health.

### Strengths and Limitations

The application of LDA modeling has clear strengths, enabling semiautomated analysis of large text corpora to readily identify barriers for data sharing. Nonetheless, some limitations present themselves; the topics identified through LDA modeling may hide rarer topics that do not have highly frequent mentions or a homogenous lexicon; for example, some *LifeInfo Survey* responses mention financial compensation (eg, vouchers), but this is not identified as a topic. In addition, texts that are linked by similar terms but are thematically different are sometimes grouped by LDA modeling; for example, HA6 included responses that require *100 percent* reassurance of certain criteria. However, these criteria span several different issues. These more granular findings may be better identified by human qualitative coding; however, this comes with its own limitations, especially for large data sets.

The *LifeInfo Survey* sample size was large, thereby facilitating the identification of important topics; however, the size of this data set was smaller than those often used for topic modeling, and responses were relatively short. As previously mentioned, those texts that were extremely short were uncategorizable by the model, which is a limitation of this methodology and data. Similarly, the survey was designed to elicit responses only from those unwilling or unsure about sharing their data. This provided benefits as it focused on the scope of topics to identify key barriers; yet, it would be insightful to obtain the opinions of those more supportive of lifestyle data sharing initiatives (which was 52.30% (2521/4820) of loyalty card holders and 70.80% (1717/2425) of health and fitness app users in our study), which should be considered in future studies. In examples where positive and negative responses are captured, it would be useful to explore a sentiment analysis approach to text mining; however, this was not relevant for our study.

Research has found LDA model results to be sensitive to model hyperparameters [44], and it is possible to use methods that optimize LDA across different α and β values. These methods were not applied in this study as trialing them increased the computational intensity of the analysis and did not provide better solutions.

Due to resource limitations, the *LifeInfo Survey* questionnaires were only available in English. This means that we are unlikely to have reached the <2% of the population who are not able to speak English. However, the *LifeInfo Survey* is overrepresented in the traditionally hard-to-reach, most deprived communities and Asian and other ethnicities [28].

### Recommended Actions

Several actions can be taken by researchers to directly address the key barriers to data sharing identified through LDA modeling, a summary of which is detailed in Textbox 1.

**Summary of recommendations to improve support for the linkage of novel consumer and lifestyle data with health records for research purposes.**

Motivation

- Provide detailed and specific information about the study purpose and benefit.

Control and consent

- Provide detailed information about research and specific opt-in mechanisms to give participants more control.

Access by others

- Provide an explicit statement that data linkage does not give all parties access to linked data. Lifestyle data will not be shared with health services, and health records will not be shared with supermarkets or technology companies.

Third-party access

- Provide reassurances that data will not be shared with third parties, such as health insurers.

Inaccurate data

- Provide acknowledgment within the study specification that data might be partial and outline mechanisms for how this will be addressed, such as data quality checks, modeling techniques, and supplementary data collection.

Non and/or infrequent use of services

- Increase participation in novel data collection and more complete use.

Data security and protection

- Put in place stringent precautions to keep data protected from hacks or data breaches.

Personal data

- Provide assurances that data will be made anonymous and nonidentifiable.

“Big Brother” and privacy invasion

- Widely used good practice and exemplar studies, which provide a clear benefit to public health, and excellent data security could help increase trust in data sharing initiatives.

Findings and recommended actions incorporate some areas we hypothesized would emerge, for example, data security and protection, but are far more comprehensive and nuanced than the existing literature reflects.

### Future Work

There is extensive scope to use LDA in future research, where free-text responses are collected in large surveys. LDA allows the detection of topics in free-text responses to be generated at a scale that is not feasible in more traditional qualitative thematic methods. Other text mining approaches, such as sentiment analysis, may be applicable where identification of positive and negative responses is important.

More work is needed to be able to further unpick concerns within topics such as *who has access to data*, where this could be context-dependent, for example, patients with a complex medical history who are worried about being judged on their lifestyle behaviors by their clinical team, and *big brother*, where it is clear that greater transparency is required regarding data being used for research purposes.

Future research could explore the utility of encouraging patients or research participants to use store loyalty cards and health and fitness apps or wearables as part of their personalized care, extending research that is already being done. This is of particular significance for those relating to the *don’t use services* topic.

There has been significant interest in the use of supermarket loyalty cards and health and fitness app data in health research, in addition to the greater availability of these data in recent years. This provides exciting opportunities to gain new insights into lifestyle risk factors for diseases through individual-linked data. The growth of this research requires that the common concerns of participants regarding ethics, data security, research aims, and personal privacy, among others, are understood so that they can be addressed by future projects. Researchers may use the findings and recommended actions shared in this paper so that greater trust can be built in practices of data linkage for health research.

### Conclusions

Analysis of the *LifeInfo Survey* responses with topic modeling techniques revealed key barriers that prevent people from willingly sharing their novel lifestyle data for health research. This large-scale public consultation provides actionable recommendations that will allow researchers using big lifestyle data to adapt their study design and provide safeguards based on expressed concerns important to the general public that are specific to novel lifestyle data and health record linkage.

## Appendix

Multimedia Appendix 1 LifeInfo questionnaire.

Multimedia Appendix 2 Supplementary information regarding data cleaning and processing steps.

Multimedia Appendix 3 Question response lengths before and after data processing.

Multimedia Appendix 4 Dendrograms of hierarchical clustering of topics produced from latent Dirichlet allocation models.

Multimedia Appendix 5 Tables showing the 20 topics produced by latent Dirichlet allocation modeling and the top 5 words per topic according to the response θ value.

Multimedia Appendix 6 Summary of texts that are not categorizable into any specific topic by the latent Dirichlet allocation model.

Multimedia Appendix 7 Graphs showing mean topic prevalence and standard error bars broken down by demographic groups.

Multimedia Appendix 8 Coded tables of multiple latent Dirichlet allocation outputs to test topic stability.

Multimedia Appendix 9 Participant information sheet.

## Abbreviations

DTM: document term matrix
LDA: latent Dirichlet allocation
