# Supplementary material for: Understanding Barriers to Novel Data Linkages: Topic Modeling of the Results of the LifeInfo Survey
Source: J Med Internet Res. 2021 May 17;23(5):e24236. doi: 10.2196/24236 (PMC8167605; doi:10.2196/24236)
Supplement: Multimedia Appendix 1 [file jmir_v23i5e24236_app1.pdf]

What do you think about researchers using your lifestyle information?

Thank you for agreeing to take part in our study. Please complete the following short questionnaire.

1. Do you use any store loyalty cards?

YES ☐
NO ☐

If yes, go to question 2, then 3.
If no, go to question 3, imagining that you might use loyalty cards in the future.

2. Do you have the following loyalty cards (please tick all that apply):

Morrison's More ☐
Sainsbury's Nectar ☐
Tesco's Clubcard ☐
My Waitrose ☐
Other please name below: ☐

3. In the future, would you give permission for the information held from store loyalty cards to be linked with your health records on the condition it were stored safely and not shared with anyone outside of the research team?

YES ☐
NO ☐
NOT SURE ☐

4. If the answer is 'no' or 'not sure' to question 3, can you tell us what (if anything) might make you change your mind in the future?

5. **Do you have access to the internet?**

YES	
NO	

6. **Do you record lifestyle or health information using wearable devices, websites on your computer or apps on your smartphone/tablet device?**

YES	
NO	

If yes, go to question 7, then 8.
If no, go to question 8, imagining that you might use lifestyle or health wearable devices, websites or apps in the future.

7. **If you answered yes to question 6, please tell us which ones you use: (please tick all that apply)?**

Myfitnesspal	
Bounts	
Strava	
Fitbit	
MyMealMate	
Calorie counter	
SHealth or Apple health	
Other please name below:	

8. **In the future, if you were sure that the information would not be shared any further and stored securely, would you give permission for health researchers to link the information held on these devices, apps and websites with your health records?**

YES	
NO	
NOT SURE	

9. **If the answer is 'no' or 'not sure' to question 8, can you tell us what (if anything) might make you change your mind in the future?**

Please tell us about yourself:

10. What is your gender?

MALE ☐
FEMALE ☐
INDETERMINATE ☐
☐

11. What is your age?

18-25 years ☐
26-35 years ☐
36-45 years ☐
46-55 years ☐
56-65 years ☐
>65 years ☐
Prefer not to say ☐

12. How would you describe your ethnicity?

BLACK/BLACK BRITISH ☐
ASIAN/ASIAN-BRITISH ☐
MIXED ☐
WHITE ☐
OTHER ETHNIC GROUP (please specify below) ☐

13. What is the first part of your postcode? For example; LS12

*this information will only identify the general area that you live in and will not locate your street.

Please complete the boxes below for

☐ ☐ ☐ ☐

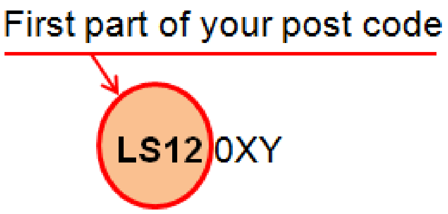

14. Which hospital clinic did you attend today?

Thank you for completing the survey.
We really appreciate you taking the time and effort to share your opinions.
