# Supplementary material for: Understanding Barriers to Novel Data Linkages: Topic Modeling of the Results of the LifeInfo Survey
Source: J Med Internet Res. 2021 May 17;23(5):e24236. doi: 10.2196/24236 (PMC8167605; doi:10.2196/24236)
Supplement: Multimedia Appendix 2 [file jmir_v23i5e24236_app2.docx]

**Appendix 2: Supplementary information about data cleaning steps**

**Stop words supplementary information**

The ‘SMART’ stop words dataset from the tm package for R was used as a base list of stop words with alterations. Information about this list of stopwords can be found here: <https://www.rdocumentation.org/packages/tm/versions/0.7-7/topics/stopwords>

The following words were removed as stop-words when single words but were retained as part of bigrams:

"big", "little", "would", "could", "why", "no", "not", "none", "need", "if", "want", "how", "sure", "about", "more", "less", "think", "use", "used", "uses", "should", "shouldnt", "wouldnt", "couldnt", "only", "know", "other", "another",,"see", "against", "allow", "anything", "associated", "available", "cant", "cannot", "consider", "definitely", "dont", "like", "dislike", "different", "maybe", "will"

The following words were retained as single words and within bigrams:

"nothing", "necessary", "appropriate".

**Misspelling and standardisation of words supplementary information**

Words that were misspelt more than once within the dataset were changed to their correct format wherever possible. These words were identified using the ‘hunspelt’ R package (<https://cran.r-project.org/web/packages/hunspell/vignettes/intro.html>). Terms that were only misspelt once were not changed.

The terms “would not”, “could not” and “should not” were standsardised to “wouldn’t”, “couldn’t” “shouldn’t”, “cannot” was standardised to “can’t”, “info” was standardised to “information”. These were changed so that equivalent word forms could be matched across unigrams and bigrams.

For the store loyalty card question “store card” was standardised to “store_card” as to not conflate the two meanings of the homonym ‘store’ within modelling e.g. ‘store’ meaning shop and ‘store’ meaning hold/retain.

**Lemmatisation supplementary information**

Lemmenisation used the textstem package within R (<https://cran.r-project.org/web/packages/textstem/textstem.pdf>) and the ‘hash_lemmas’ dictionary of lemmas from the ‘lexicon’ package (<https://www.rdocumentation.org/packages/lexicon/versions/0.7.4/topics/hash_lemmas>)

The following alterations were made:

The words “don’t”, “could”, “more”, “bit”, “boots”, and numeric terms were not lemmatised. Nor was the word “stored” as this distinguished between store as a verb (e.g. stored data) and store as a noun/adjective (e.g. the store or store card.)
