# Supplementary material for: Understanding Barriers to Novel Data Linkages: Topic Modeling of the Results of the LifeInfo Survey
Source: J Med Internet Res. 2021 May 17;23(5):e24236. doi: 10.2196/24236 (PMC8167605; doi:10.2196/24236)
Supplement: Multimedia Appendix 3 [file jmir_v23i5e24236_app3.docx]

**Appendix 3: Graphs showing response lengths of analysed texts before removal of stop words and after data cleaning and removal of stop words**


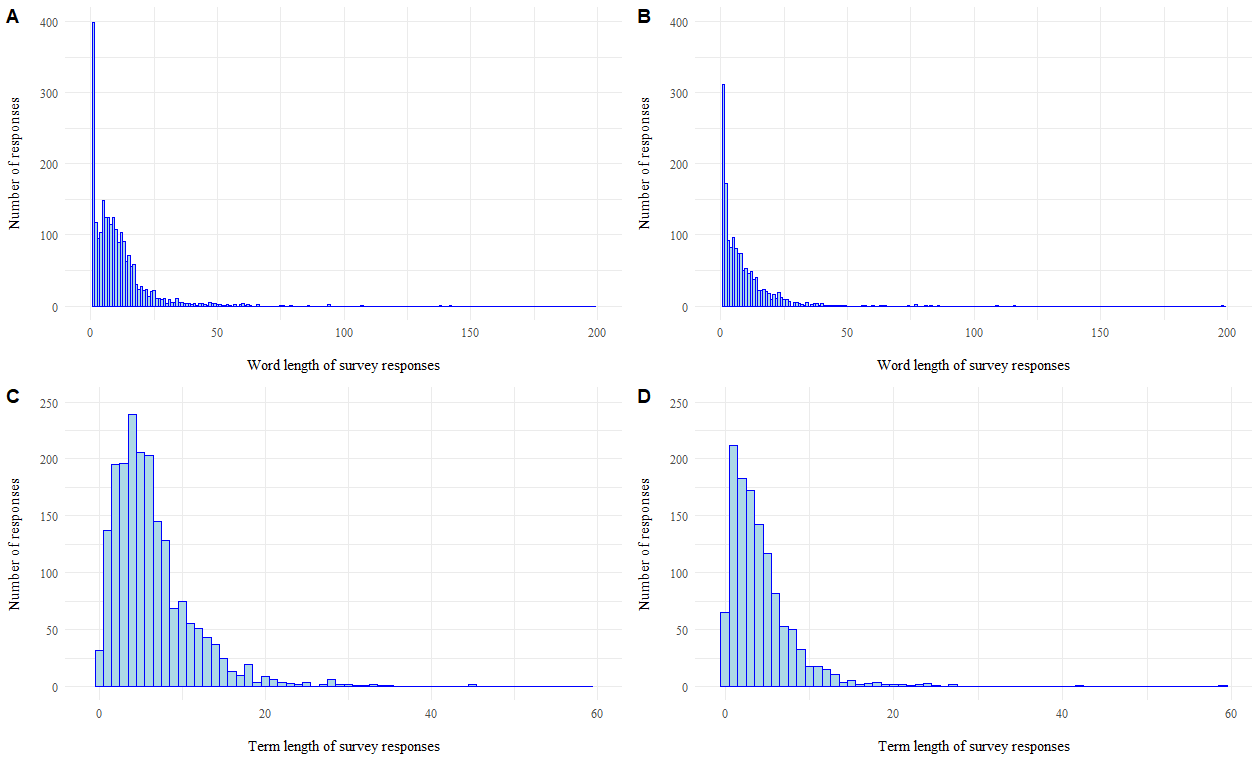


A: Frequency of word lengths of survey responses before data cleaning and removal of stop words – store loyalty card question (N= 2325)

B: Frequency of word lengths of survey responses before data cleaning and removal of stop words – health and fitness app question (N=1516)

C: Frequency of term lengths of survey responses after data cleaning and removal of stop words store loyalty card question (N= 1930)

D: Frequency of term lengths of survey responses after data cleaning and removal of stop words health and fitness app question (N= 1206)
