# Supplementary material for: Understanding Barriers to Novel Data Linkages: Topic Modeling of the Results of the LifeInfo Survey
Source: J Med Internet Res. 2021 May 17;23(5):e24236. doi: 10.2196/24236 (PMC8167605; doi:10.2196/24236)
Supplement: Multimedia Appendix 4 [file jmir_v23i5e24236_app4.docx]

**Appendix 4**: Dendrograms of hierarchical clustering of topics produced from LDA models

Dendrogram of 20 topics created from LDA for the LifeInfo **store loyalty card question** with 9 categories created through hierarchical clustering shown in blue


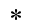

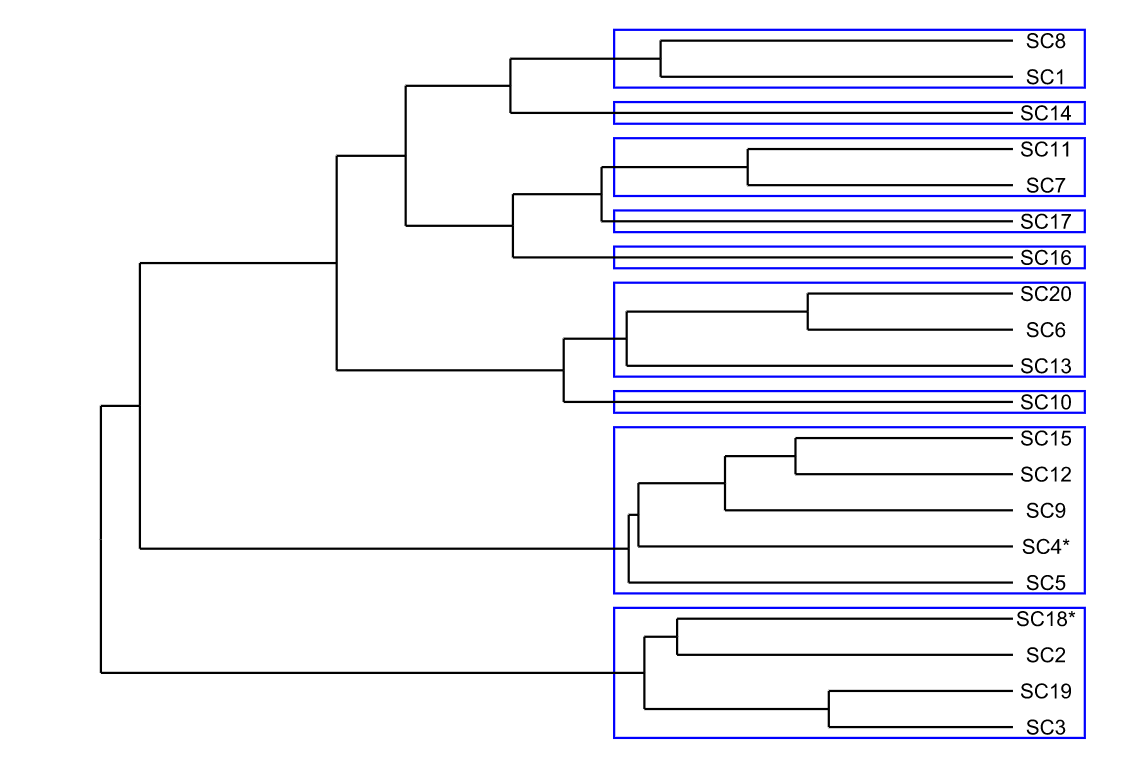


Dendrogram of 20 topics created from LDA for the LifeInfo **health/fitness app question** with 10 categories created through hierarchical clustering shown in blue.


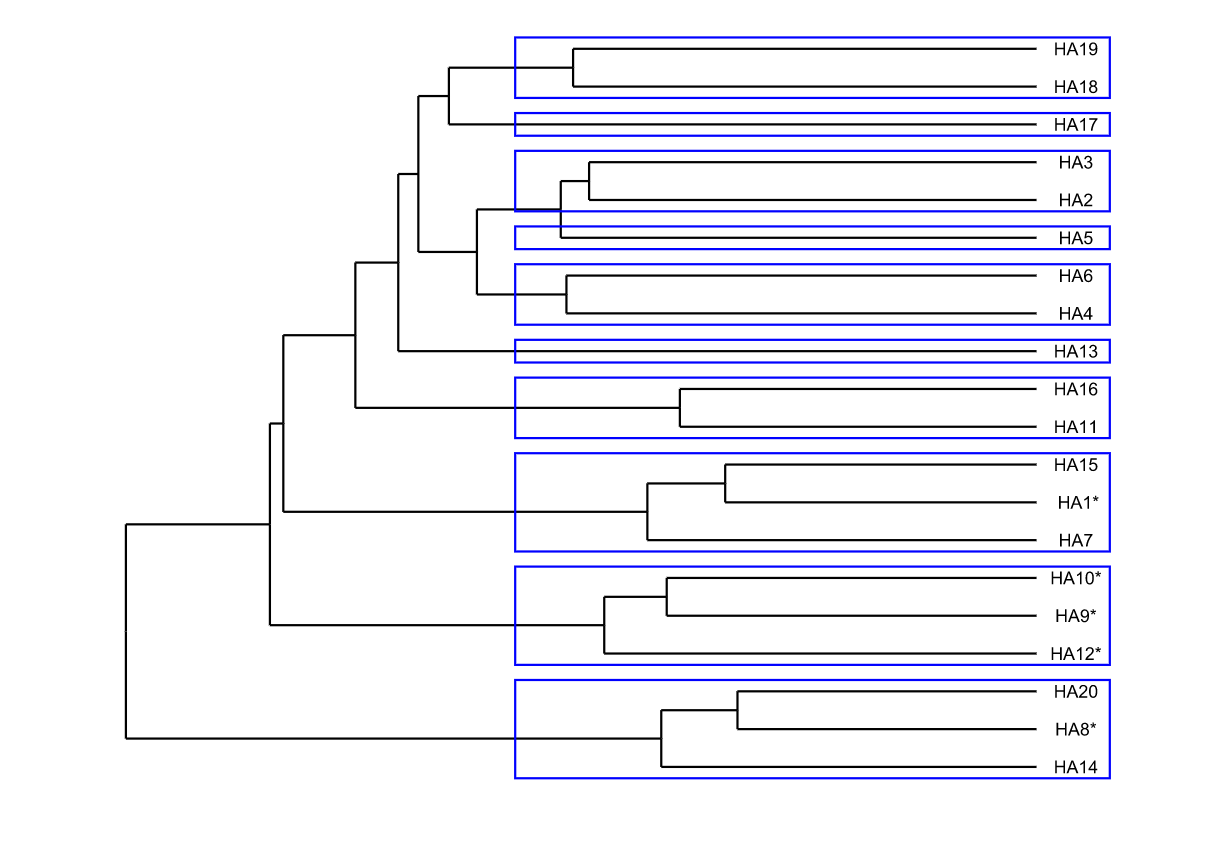


These dendrograms of hierarchical clustering of topics produced from LDA models show the topic groupings that are created with each iteration of group clustering – matching the two most similar groups together. From left to right each of the forks shows potential topic groupings from their minimum (2) to their maximum (20). The number of clusters was chosen by moving from left to right and observing if the proposed split broke a group of topics that thematically are similar (e.g. would the proposed split break a sensible category of ‘data security’ into categories that are not substantively different?). This process fixed the number of thematic groups, shown as the blue squares, and then manual reassignment of topics that did not align with their group was undertaken. Those topics that have been manually reassigned in the topic tables are marked with asterisk.
