# Supplementary material for: Understanding Barriers to Novel Data Linkages: Topic Modeling of the Results of the LifeInfo Survey
Source: J Med Internet Res. 2021 May 17;23(5):e24236. doi: 10.2196/24236 (PMC8167605; doi:10.2196/24236)
Supplement: Multimedia Appendix 5 [file jmir_v23i5e24236_app5.docx]

**Appendix 5:** Tables showing the 20 topics produced by LDA modelling and top five words per topic according to response theta value.

**Store loyalty card question**

| *Topic* | *Top five responses associated with topic according to document theta value* | *theta* |
| --- | --- | --- |
| **Theme: Wouldn’t change mind** | |  |
| *Wouldn’t change mind* | | |
| SC14 | I don’t think anything would change my mind. I don’t want strangers to see my transactions. | 0.81 |
|  | Nothing would make me change my mind | 0.79 |
|  | Nothing would make me change my mind. | 0.79 |
|  | Can't think of anything likely to make me change my mind - I am very opposed to this | 0.79 |
|  | Don't want to. Nothing will make me change mind | 0.79 |
| **Theme: Store loyalty card/ don’t use store card** | | |
| *‘Store loyalty card’/ don’t use* | |  |
| SC16 | Store loyalty card+ health info are totally different. No need to be linked | 0.78 |
|  | I have loyalty cards but not from any stores so I don't think they are what you are looking for. | 0.76 |
|  | Currently don’t use store loyalty cards | 0.76 |
|  | Nothing. What on earth has a store loyalty card go to do with my health?! | 0.76 |
|  | I don't use any store cards and I am not interested in using them . | 0.76 |
| **Theme: Privacy and ‘Big Brother’** | | |
| *Big brother/ nanny state* | | |
| SC8 | Nothing, I feel it would be an invasion of privacy if you want or don't want to tell doctor what you eatis still your choice | 0.83 |
|  | Not interested to a "big brother is watching" on all aspects of my life | 0.76 |
|  | Big brother/nanny state | 0.68 |
|  | I was inclined to say yes, but on second thoughts I am not so sure. I wouldn't want the GP to know everything I purchased and then bring it up if he feels I need to change something. It's useful, but I feel my privacy is threatened | 0.68 |
|  | Feels like a bit intrusive to personal life | 0.68 |
| *Privacy and cold calling* | | |
| 1 | I don't know how they have any connection with each other+ would be concerned about confidentiality breaches | 0.76 |
|  | Notsure why this would be required. Already receive enough cold calls | 0.73 |
|  | Concern about confidentiality and getting constant emails etc. | 0.68 |
|  | I don't want cold calling. It won't be safe | 0.62 |
|  | Guarantees that there will be no phone calls texts or emails | 0.62 |
| **Theme: Personal data sharing and access by others** | | |
| *Concerns about linkage and insurance* | | |
| SC2 | I don’t like the idea of so much information being linked, and I think that it potentially gives someone too much knowledge about me and my life/habits that could be used against me. | 0.84 |
|  | This information could have all sorts of repurcussions E. G. Health insurance and so I would not ever agree to it. | 0.76 |
|  | I like to think we are in a 'free' society. So, no I wouldn't like to give permission to have more bodies knowing about what I do.I think it's bad enought the privacy policies technologycopmanies how have | 0.74 |
|  | Guarantee it did not affect my life or health insurance. If I day | 0.73 |
|  | If I was sure that my health insurance Would not use any of this info against me. | 0.68 |
| *Data access and others* | | |
| SC19 | I do not want private companies to have access of my personal health info | 0.85 |
|  | Only if it can be that information will no be passed on to any 3rd parties as has happened in the past | 0.81 |
|  | Don't want unqualified individuals to have access to my personal information. If I had assurance that only professionals had access to this information then I may reconsider. | 0.78 |
|  | Unsure og other people having access to private information | 0.76 |
|  | There is already too much information collected and stored about people and too many people trying to tell us how we should live | 0.76 |
| *Don’t want to share personal information* | | |
| SC3 | Nothing, I don't want my personal information being shared with any retailer. | 0.83 |
|  | I don't want my personal information to be shared across different departments/stores | 0.79 |
|  | It is personal information and there needs to be a limit on information sharing | 0.79 |
|  | Nothing - don't like the fact that everything about me is shared info. | 0.76 |
|  | Don't really want personal information shared | 0.76 |
| **Theme: Data inaccuracy** | | |
| *Data inaccuracy and bias* | | |
| SC7 | No, I would be very dubious about the new then turning around in the future and refusing care to patients based on purchases. In my case it would not be a reflection of my shopping as I buy all my fruit and veg at a farm shop is not a supermarket. Taking my data would provide misleading associations. | 0.85 |
|  | What I buy does not necessarily reflect what I eat. Eg, I could be buying alcohol or chocolates as gifts for people. I would be interested in how you would get around this. | 0.83 |
|  | Don't have a problem with there being access to the data but my concern would be about the bias that this could cause as the majority of people are likely to be buying food for others e.g. I personally don't eat meat products but as my husband and son do I will be buying meat. I also do a significant amount of shopping at Aldi, local greengrocers, farm shops etc so therefore the products bought at stores would not be representative of mine or my families diet | 0.82 |
|  | I think it would be of limited use. What I buy at the supermarket isn't necessarily eaten by me, also I don't use the supermarket exclusively for food. So you could look at My morrisons data and conclude I don't eat meat or veg, when actually I buy them from the butcher/green grocer. | 0.82 |
|  | Because tthe cards indicate products I buy for others inside and outside family and is not indicative of my personal consumption or lifestyle | 0.81 |
| **Theme: Data security and protection** | |  |
| *Don’t trust organisations with data* | | |
| SC15 | Nothing, don't trust large organisations or corporations to protect my personal data | 0.84 |
|  | I trust nhs not to use the data or share but there is no guarantee with hacking | 0.84 |
|  | Don't trust private organisations to use my data, safety | 0.79 |
|  | Don't trust the robustness of security of computer systems of nhs(or other large organisations). Lot's of data stored not keen on letting an organisation hold on to this | 0.78 |
|  | Nothing - I want a limit on how my data is shared and do not trust corporations or the NHS to be secure in their practices. | 0.76 |
| *Data protection* | | |
| SC12 | Waitrose yes, Nectar no. Nectar support right wing newspapers, I would not want them having access to my health data | 0.76 |
|  | More secure data protection | 0.73 |
|  | More secure data protection | 0.73 |
|  | Privacy + data protection issues | 0.68 |
|  | If it was anonymous then yes but wouldn’t want the data to be linked to be identifiable | 0.68 |
| *Data security* | | |
| SC9 | No. Becuase the risk of data breach is increased and I would like to remain anonymous. | 0.76 |
|  | Worry about data breaches/ commercial opportunities being exploited by others | 0.76 |
|  | Better security (there's too much in the news about breaches of data security) | 0.76 |
|  | Improvement in information security, too many data leaks of perfonal information in recent times | 0.73 |
|  | Increased security of data and concrete assurance this would not be breached | 0.73 |
| *Guaranteed data safety/security* | | |
| SC5 | System need to be 100% secure. I question governamce issues of scr-not 100% safe | 0.86 |
|  | Would prefer my records to remain secure and until this was guaranteed then would prefer security | 0.79 |
|  | If I could be convinced that it would be secure (and this would continue indefinately) and if I could be convinced of the benefits | 0.76 |
|  | Not convinced of NHS' ability to maintain confidentiality of their records, and to transfer data securely to third parties. | 0.76 |
|  | Such data collection seems a bit intrusive. Not 100% convinced it would always remain anonymous. | 0.74 |
| **Theme: Understanding benefit and details** | | |
| *Don’t understand benefit* | | |
| SC20 | I don't understand why these would need to be linked- if I understand i=I may consider again | 0.81 |
|  | I don't understand why the two would need to be linked when they are totally unrelated | 0.79 |
|  | I don't understand why this would be necessary or beneficial | 0.76 |
|  | I don't understand why it needs to be linked | 0.73 |
|  | I don't understand why it would be of benefit | 0.73 |
| *Require demonstratable benefits* | | |
| SC6 | If you could explain what benefit or direct link this would have and why it is appropriate? | 0.76 |
|  | I'm not sure what my personal/political position is on privacy vs benefit / public benefit at this point. | 0.68 |
|  | Would need to explain the benefit of this happening. | 0.62 |
|  | I would need to see what the benefit would be | 0.62 |
|  | I'm not sure as to how the two would link +benefit me in any way | 0.62 |
| *Require reassurance* | | |
| SC4* | If the data fed into important public health or similar research and was not used to further commercial gains by these giants of commerce | 0.85 |
|  | If I knew about the specific research study in advance and had consented to take part, that would be OK | 0.79 |
|  | May in specific studies- would want to give specific consent, not bnaket consent | 0.79 |
|  | I would be happy as long as I was informed of the purpose of the research | 0.76 |
|  | If I knew what the specific study was and that permission was not blanket permission for researchers to use as they please when they please | 0.76 |
| *Require more information* | | |
| SC18* | I would like to know exactly what information would be used, what information would be made available and for what purpose | 0.84 |
|  | Would need to know more info e.g. why they would want to know | 0.81 |
|  | I would want to know why you wanted the info +what you would do with it, voucher for signing | 0.79 |
|  | Need more info on what info was being gathered + how it might be used | 0.79 |
|  | I would need more information detailing what this info would be used for | 0.79 |
| **Theme: Health Records shouldn’t be linked** | | |
| *Health record should not be linked* | | |
| SC17 | Think health records should be private so nothing | 0.79 |
|  | Health records should be kept in health care | 0.76 |
|  | Don't think health records should be open to companies | 0.76 |
|  | Nothing, health records are private | 0.76 |
|  | Why would I want Tesco knowing my health records? | 0.73 |
| *Shopping habits and health shouldn’t be linked* | | |
| SC11* | Nothing - healthcare isn't a commercial enterprise and PLC's won't be interested in part nationalising any part of their business | 0.73 |
|  | Feel my healthcare is an issue for health professionals not for commercial exploitation | 0.73 |
|  | I'd like to know a little more on what they would be checking. Also i don't do my full weekly shop at one store | 0.68 |
|  | Often shop for several people- may be confusing. Do not have a health conditions at present | 0.68 |
|  | My shopping habits have nothing to do with my medical conditions | 0.68 |
| **Theme: Don’t understand reason/relevance of data linkage** | | |
| *Unsure of reason for linkage* | | |
| SC13 | I don't see a supermarket would need my medical records | 0.79 |
|  | Don't see how store could benefit by seeing my sons medical records | 0.73 |
|  | I don't see how it is relevant to my medical notes | 0.68 |
|  | I can't see how that is relevant to health | 0.68 |
|  | Explain why they would need my medical information | 0.68 |
| *Don’t see reason for linkage* | | |
| SC10 | Cannot really see the connection, but if a good reason was provided, I would reconsider. | 0.79 |
|  | I don't see the relevance of linking 2 very different providers | 0.76 |
|  | Do not see any reason why they should be linked | 0.76 |
|  | I just don't see the relevance in the 2 being linked | 0.73 |
|  | Nothing- there is no reason to link the two | 0.73 |

**Health/fitness app question**

| *topic* | *Top five responses most associated with topic according to document theta value* | *theta* |
| --- | --- | --- |
| **Theme: Nothing would change mind** | |  |
| *Wouldn’t change mind* | | |
| HA17 | Nothing would make me change my mind on this issue. | 0.81 |
|  | As above. At this moment I can not see anything that would make me change my mind | 0.81 |
|  | Nothing would change my mind. If I need help I will ask for it | 0.79 |
|  | Nothing would make me change my mind | 0.79 |
|  | Nothing would make me change my mind. | 0.79 |
| **Theme: ‘Store loyalty card’/ don’t use store card** | | |
| *‘Device’ and don’t use* | | |
| HA16 | I'm not going to use a wearable device in the future | 0.73 |
|  | Wearable devices maybe but not other apps | 0.68 |
|  | Don't use devices+ probably won't in future | 0.68 |
|  | Doesn't wear devices and can't see changing | 0.62 |
|  | If wearable device was supplied by nhs as part of treatment | 0.62 |
| *‘App’/’website’ and don’t use* | | |
| HA11 | While there might be a large cohort who use all fitness gadgets, there will always be a minority who can take reasonable lifestyle modifications without the use of app. | 0.76 |
|  | I didn't correctly use apps so not applicable to me at present | 0.76 |
|  | There would be no point as I don't use loyalty cards or fitness apps unless you needed a controlled sample of non-users | 0.73 |
|  | Most unlikely I would be using a fitness app | 0.68 |
|  | Don't use lifestyle websites. Apps etc. | 0.62 |
| **Theme: ‘Big Brother’ and privacy invasion** | | |
| *‘Big Brother’* | |  |
| HA3 | My concern is that people don't understand the complexities of illness. By seeing that I go for a walk at lunchtime, the assumption is likely to be 'she's healthy'. But it doesn't tell you how ill I am in the mornings etc. The general idea makes me uncomfortable as if there's a degree of judgement. It's a tricky thing to put my finger on to explain! I guess I feel like i'm already 'under the nhs system' enough and wouldn't want to feel every area of my life is out of my control and being watched by an institution that already makes me feel like I have no autonomy. | 0.84 |
|  | Make watches more comfortable and affordable (the good smart watches that is). Also, we can't wear watches at work | 0.76 |
|  | If I thought it would help people live better , healthier lifes | 0.68 |
|  | I'm well aware of what healthy living is so don't feel that I need any outside support. | 0.68 |
|  | Again I feel it looks too much like big brother | 0.68 |
| *Privacy invasion and safety/security of data* | |  |
| HA2 | Very little I am not reassured by the suggestion that data is 'stored securely'; I cannot see how this can be guaranteed to me. The obvious example is that you ask for my gender, age and post code. I have been truthful abt the first 2. Had I also given you my correct post code, then I would have been immediately identifiable - there are very few houses with that postcode, and I am the only person fitting the other 2 parameters | 0.81 |
|  | As above partly because I don't believe you would store it securely and partly because of the potential for control and sureveillance | 0.73 |
|  | Privacy issues- difficult to trust that it will be 'stored securely' | 0.73 |
|  | Don't know, I feel it is a massive invasion of privacy | 0.68 |
|  | Nothing. I feel it would be an invasion of privacy | 0.68 |
| **Theme: Personal information sharing** | | |
| *Information would be shared* | | |
| HA20 | You can never be sure that the info wouldn't be shared or hacked | 0.73 |
|  | If I know exactly who my info will be shared with | 0.73 |
|  | I don't currently use one but if I did I would have to think about the information on there to be shared. | 0.68 |
|  | Nothing, can't be sure information would not be sold or shared with others | 0.68 |
|  | It may be said now that info. Wouldn't be shared but now -a- days there's no certainity | 0.68 |
| *Information is personal* | | |
| HA14 | Nothing, this information is for my personal use. Access to my private devices can lead to security risks | 0.79 |
|  | I don't want my personal information to be accessed by other parties | 0.76 |
|  | Don't want unqualified individuals to have access to my personal information. If I had assurance that only professionals had access to this information then I may reconsider. | 0.74 |
|  | It's private info for personal use | 0.73 |
|  | Private info for personal use | 0.73 |
| **Theme: Who has access to the data?** | | |
| *Data access by insurance/private companies* | | |
| HA15 | If the information was available only to the research team, and not to others, e.g. Insurance companies, mortgage companies, even the medical team | 0.76 |
|  | My only concern would be if the data stored were, in the future, used by insurance companies to tailor a premium based on my lifestyle especially if it were not favourable - like car insurancers are promoting the use of boxes in cars to monitor driving habits. | 0.65 |
|  | Information on how research will be used. Unsure re use results by e.g. insurance companies etc. | 0.64 |
|  | As long as it wasn't shared with insurance companies or used against me | 0.62 |
|  | Not always relevant- I don't wear my fitbit every day | 0.59 |
| *‘Health records’ and linkage* | | |
| HA7 | These organisations are profit making entities. I do not want them to have access to my health records, untrustworthy | 0.76 |
|  | It's my information for me. If I want my doc to know it I'll tell him/her or put it on my health record myself | 0.73 |
|  | I don't want anything linked to my health records | 0.73 |
|  | My health records should already have upto date information ie. Hight, weight, bmi etc. | 0.68 |
|  | Nothing. I don't; t want my health record linking to my life | 0.68 |
| **Theme: Data inaccuracy** | | |
| *Inaccurate data and partial use* | |  |
| HA5 | I don't necessarily think it would be accurate for casual users. For example, mine shows a terrible step count, but that's because I don't hold my phone while playing netball, long walks etc!! | 0.83 |
|  | Not currently because although I record my daily steps using shealth, I do not bring my phone with me when exercising, which would mean I wouldn't be sharing the full picture regarding my exercise. If I started to capture all of my physical activity (I don't currently as I don't want to hold my phone whilst running but I could get some kind of pouch to hold my phone), then I'd change my mind. | 0.72 |
|  | It's not accurate. For example I used to use mapmy run but now I don't- but I still run as much+ didn't log everything , so it wouldn't reflect my lifestyle | 0.68 |
|  | Too personal and again not a full reflection of my fitness activity | 0.62 |
|  | The information from loyalty store cards and activity recorders is not an accurate reflection of health of an individual. For instance I might buy biscuits but they are not for me they are for the window cleaner, I buy alcohol for my husband (I am teetotaller), etc. My activity monitor is not always with me so my activity is usually more than is recorded on the device so is not accurate. I don't think either of these methods is an accurate reflection of an individual's health. | 0.60 |
| **Theme: Data security and protection** | | |
| ‘Not sure’ and security | | |
| HA19 | Not sure about how secure it would be | 0.73 |
|  | Not sure how secure this would be | 0.68 |
|  | It would have to be very secure, not sure about that | 0.62 |
|  | Not sure if I would come across this situation | 0.62 |
|  | Since I do not [illegible] not sure if I'm willing to have someone else to [illegible] | 0.62 |
| *Don’t trust data security* | | |
| HA18 | Don't trust info is secure | 0.68 |
|  | Nothing. I don’t trust this sort of thing | 0.68 |
|  | Nothing is secure enough to prevent fraud and theft actions | 0.68 |
|  | Nothing is ever secure on a computer data base no matter how many passwords are on it | 0.68 |
|  | Nothing, don't trust computers | 0.68 |
| *Data protection against sharing* | |  |
| HA10* | You cant ever be sure data is not shared any further even if embedded in law. Also data breach is increased so no. | 0.83 |
|  | Data protection and concerns if my data could be mis-used. | 0.76 |
|  | Look at yesterday's news! Data from these also include geographic data as well as activity. I do not wish to share this data. | 0.76 |
|  | My data is my data not to be shared unless a request is made that would be helpful to me | 0.73 |
|  | Can't be assured of data protection between the various systems | 0.68 |
| *Requires data security* | | |
| HA12* | Concerned about data security | 0.73 |
|  | Unsure about security & matters for use of the data | 0.73 |
|  | Even tough assured of security would need to have more confidence in internet safety. | 0.64 |
|  | Worried about security | 0.62 |
|  | Complete assurance about matters of security and confidentiality. | 0.62 |
| **Theme: Understand research purpose and process** | | |
| *Depends on assurances and purpose* | | |
| HA6 | THERE IS NO 100% guarantee INFO WOULD NOT BE HACKED- LOOK AT BANKS ETC. | 0.64 |
|  | It would depend on the purpose of the use by researchers. | 0.62 |
|  | Unsure that devices are 100% safe | 0.62 |
|  | 100% assurance THAT NO BREACHES WOULD OCCUR | 0.62 |
|  | It would depend on what kind of research was being carried out | 0.62 |
| *Consent to specific research* | | |
| HA4 | I would be happy if the information was only used for research purposes | 0.79 |
|  | If it was for a specific research project and have consent forms and it was just for short time period I would consider | 0.76 |
|  | Has to be linked to a condition or specific research project with additional approval provided in advance | 0.76 |
|  | If I knew what the specific study was and that permission was not blanket permission for researchers to use as they please when they please | 0.73 |
|  | I may give permission for specific researchers but not blanket permission | 0.73 |
| *Understanding how and why data would be used* | | |
| HA9* | Again do not understand why and what the benefits would be how it would be used | 0.76 |
|  | My use is sporadic and inconsistent. I would need to know if this kind of use has any benefits or would lead to messy data and any conclusions based on limited data. | 0.74 |
|  | I would need to be briefed about the purpose and ways in which the data would be processed. I would need to be sure it's not for commercial use | 0.73 |
|  | Information on how and why the data would be used | 0.62 |
|  | Again, cleared about how this would be used | 0.62 |
| *Requires more information* | | |
| HA8* | I would need huge assurance that the information would be totally anonymous and would not be sold or made available to third parties. | 0.76 |
|  | Would need information as to what the information would be needed for | 0.76 |
|  | Would need more detailed information regarding data base etc. | 0.73 |
|  | Would need more info as to why it is needed | 0.73 |
|  | More information about how it will be used and why | 0.73 |
| *Benefits to health* | | |
| 1 | I'd like to know why they'd want to link the information and what benefit/effect it would have on my health care treatment. | 0.79 |
|  | I would like to know that the researchers were complying with a code that meant they were not interested in my individual results, just the benefits of linking more generally. | 0.79 |
|  | Health benefits and recommended action of health professionals | 0.76 |
|  | Possible as health researchers are in the similar family as lifestyle and health so can see the link | 0.76 |
|  | I may choose to wear it intermittently, I provide to health care professionals if I consider it necessary | 0.68 |
| **Theme: Same answer as question 4 (store loyalty card question)** | |  |
| *Same answer as Q4* | | |
| HA13 | Nothing - please see answer to question 4. | 0.76 |
|  | See answer to question 4. | 0.73 |
|  | Essentially this is the same answer as question 4 | 0.68 |
|  | See answer to Q4 | 0.62 |
|  | See answer to q4 | 0.62 |
