# Supplementary material for: Understanding Barriers to Novel Data Linkages: Topic Modeling of the Results of the LifeInfo Survey
Source: J Med Internet Res. 2021 May 17;23(5):e24236. doi: 10.2196/24236 (PMC8167605; doi:10.2196/24236)
Supplement: Multimedia Appendix 6 [file jmir_v23i5e24236_app6.docx]

**Appendix 6**: Texts that are not categorisable into any specific topic by LDA model

| **Store loyalty card question** | | |
| --- | --- | --- |
|  | Number of Texts (N= 1930) | Proportion of Total |
| ‘Uncategorisable’ texts | 270 | 14.0% |
| ‘Categorisable’ texts | 1660 | 86.0% |
| **Health/fitness app question** | | |
|  | Number of Texts (N= 1206) | Proportion of Total |
| ‘Uncategorisable’ texts | 416 | 34.5% |
| Categorisable texts | 790 | 65.5% |

Text that are ‘uncategorisable’ have equal theta values of 0.05 across all topics and, therefore, cannot be categorised as belonging to one topic more than any others.

Texts that are ‘categorisable’ have one or more topics that have theta values that are higher than those for other topics, therefore, can be categorised as belonging to one or more topic.
