# Supplementary material for: Understanding Barriers to Novel Data Linkages: Topic Modeling of the Results of the LifeInfo Survey
Source: J Med Internet Res. 2021 May 17;23(5):e24236. doi: 10.2196/24236 (PMC8167605; doi:10.2196/24236)
Supplement: Multimedia Appendix 7 [file jmir_v23i5e24236_app7.docx]

**Appendix 7: Graphs showing mean topic prevelance and standard error bars broken down by demographic groups**


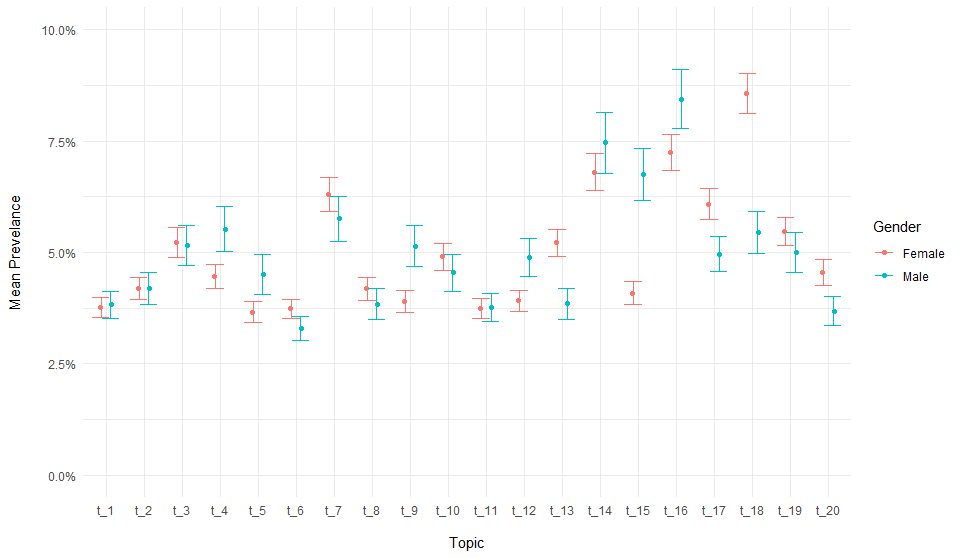


Figure A: Mean topic prevalence (%) and standard error bards for topics created by LDA modelling for the store loyalty card question by gender.


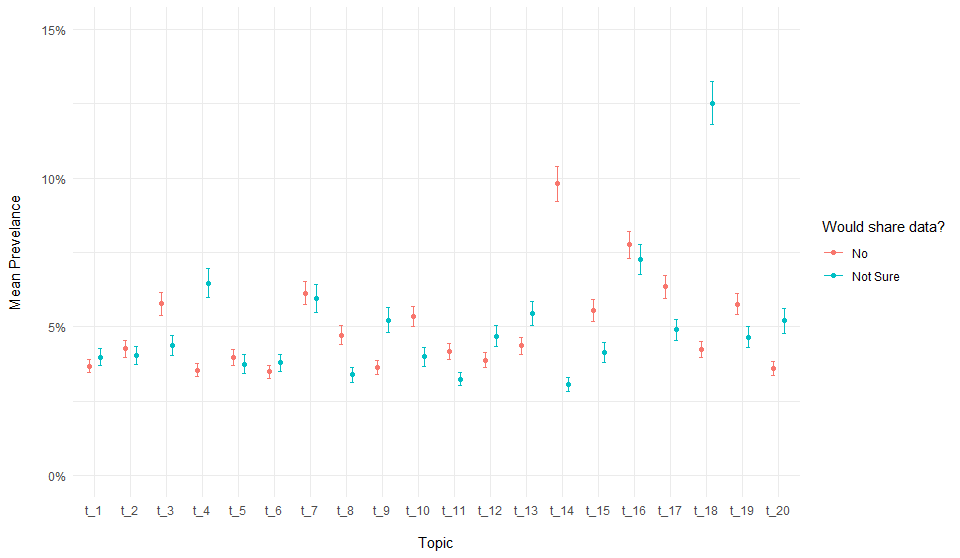


Figure B: Mean topic prevalence (%) and standard error bards for topics created by LDA modelling for the store loyalty card question by those who responded they would not share their data and those who responded not sure.


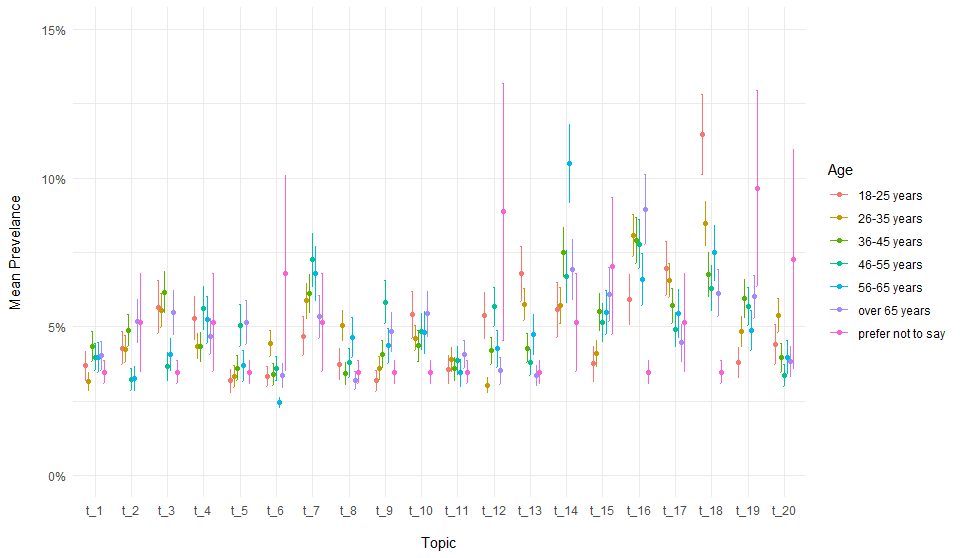


Figure C: Mean topic prevalence (%) and standard error bards for topics created by LDA modelling for the store loyalty card question by age categories.


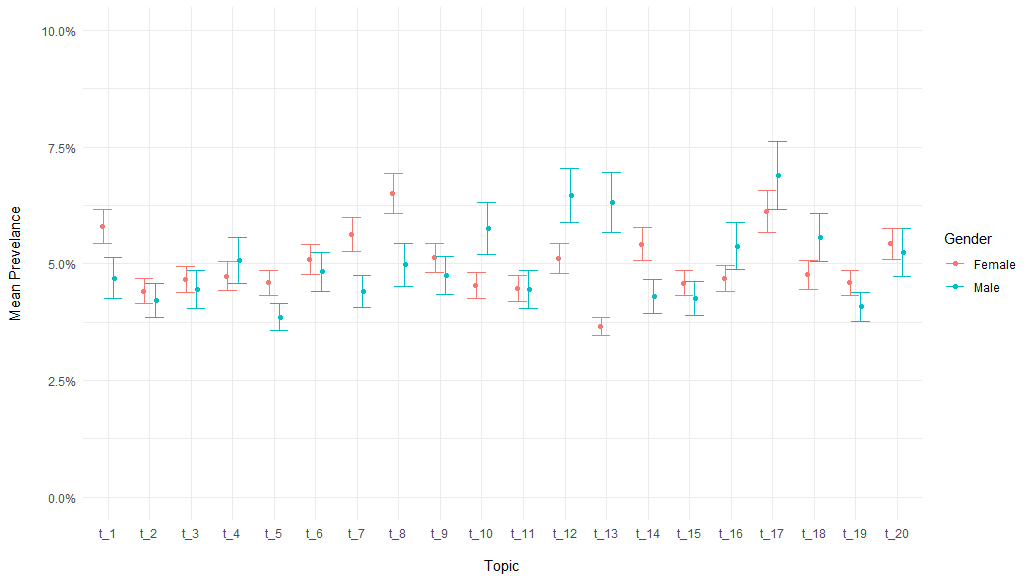


Figure D: Mean topic prevalence (%) and standard error bards for topics created by LDA modelling for the health and fitness app by gender.


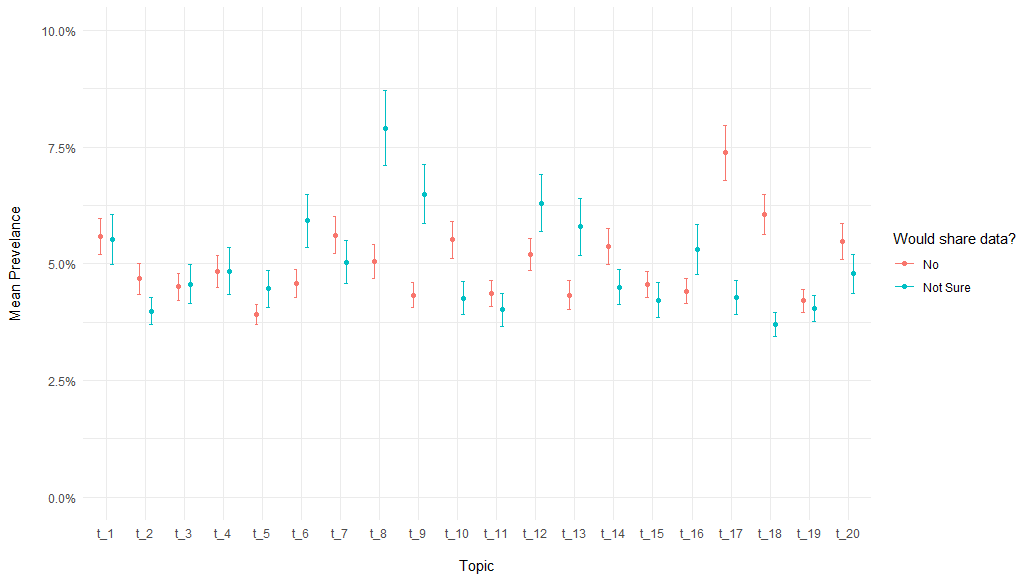


Figure E: Mean topic prevalence (%) and standard error bards for topics created by LDA modelling for the health and fitness app question by those who responded they would not share their data and those who responded not sure.


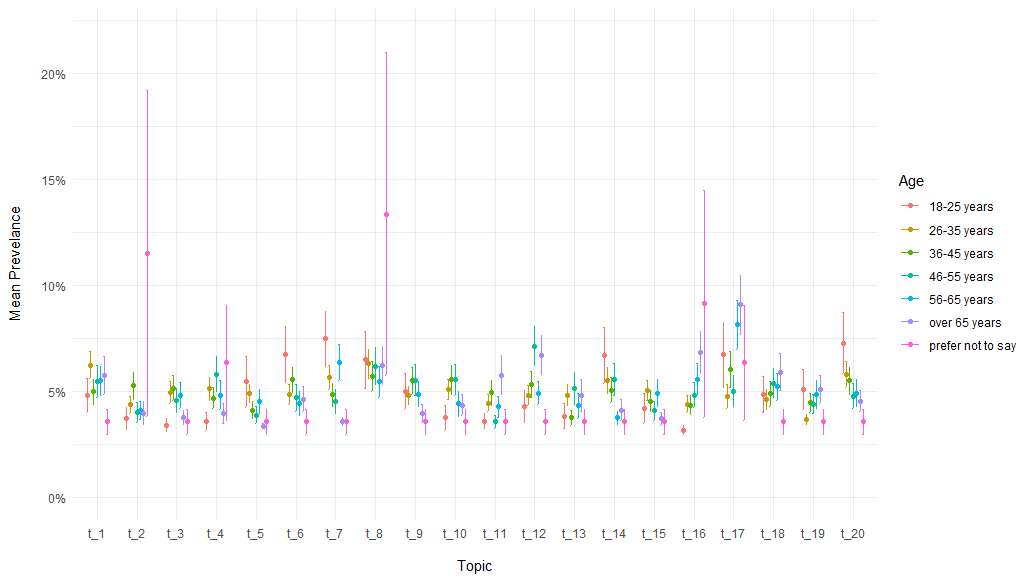


Figure F: Mean topic prevalence (%) and standard error bards for topics created by LDA modelling for the health and fitness app question by age categories.
