# Supplementary material for: Understanding Barriers to Novel Data Linkages: Topic Modeling of the Results of the LifeInfo Survey
Source: J Med Internet Res. 2021 May 17;23(5):e24236. doi: 10.2196/24236 (PMC8167605; doi:10.2196/24236)
Supplement: Multimedia Appendix 8 [file jmir_v23i5e24236_app8.docx]

**Appendix 8:** Outputs of Multiple LDA outputs to tests Topic Stability

The topics created across multiple LDA runs for both the store loyalty card question and the health and fitness app question indicate that topics are relatively stable. Topics can be categorised into the same themes as the final model included within the manuscript, as indicated by the colour coding within the tables, and although some differences do apply highly similar topics are created across all runs.

Store loyalty card question

Thematic colour key:

|  | Health records shouldn’t be linked |
| --- | --- |
|  | Big brother and privacy invasion |
|  | Data accuracy |
|  | Store loyalty card/ don’t use store loyalty card |
|  | Understanding purpose and process |
|  | Nothing would change mind |
|  | Personal information sharing and access by others |
|  | Don’t understand reason for data linkage |
|  | Data security and protection |

Topic model 1

| *topic* | *coherence* | *prevalence* | *top_terms_phi* |
| --- | --- | --- | --- |
| t_1 | 0.34 | 7.12 | card, loyalty, loyalty_card, store, store_card, store_loyalty, health, dont_use, card_health, link |
| t_2 | 0.13 | 5.83 | data, trust, share, data_protection, protection, nhs, dont_trust, data_would, secure, organisation |
| t_3 | 0.06 | 4.71 | research, data, depend, purpose, specific, would_depend, team, anonymise, study, commercial |
| t_4 | 0.13 | 5.76 | benefit, understand, would_need, why_would, dont_understand, link, understand_why, not_sure, necessary, benefit_would |
| t_5 | 0.09 | 4.17 | shop, buy, supermarket, relate, family, diet, healthy, lifestyle, food, aldi |
| t_6 | 0.04 | 3.80 | give, information, health, insurance, access, care, health_insurance, affect, provide, impact |
| t_7 | 0.26 | 5.77 | health, record, health_record, link, link_health, nothing, why_would, store, private, not_sure |
| t_8 | 0.47 | 6.81 | change, mind, change_mind, nothing, would_change, nothing_would, dont_think, wouldnt_change, make, nothing_change |
| t_9 | 0.07 | 4.37 | personal, information, idea, privacy, dont_like, like_idea, personal_information, invasion, invasion_privacy, personal_data |
| t_10 | 0.03 | 3.48 | risk, future, require, market, confidentiality, privacy, call, email, guarantee, advertise |
| t_11 | 0.08 | 4.45 | stored, information, safe, secure, 100, 100_percent, percent, not_sure, convince, information_stored |
| t_12 | 0.09 | 3.54 | medical, record, medical_record, information, medical_information, prefer, doctor, confidential, store, history |
| t_13 | 0.07 | 4.47 | relevant, health, shop, not_sure, habit, shop_habit, relevant_health, link, nothing, not_relevant |
| t_14 | 0.05 | 4.38 | information, private, company, access, people, detail, worry, sell, hold, worry_about |
| t_15 | 0.20 | 4.02 | feel, make, big_brother, brother, choice, good, dont_feel, thing, feel_like, state |
| t_16 | 0.08 | 5.12 | reason, dont_see, relevance, link, cant_see, reason_why, see_relevance, nothing, see_why, point |
| t_17 | 0.11 | 4.16 | buy, purchase, eat, people, make, food, family, product, item, thing |
| t_18 | 0.10 | 7.58 | information, more_information, would_use, information_would, would_need, would_want, need_know, information_use, know_why, want_know |
| t_19 | 0.11 | 5.02 | security, data, concern, concern_about, data_security, privacy, assurance, breach, would_concern, confidentiality |
| t_20 | 0.12 | 5.44 | share, information, information_share, personal, share_information, dont_want, personal_information, nothing, want_information, detail |

Topic model 2

| *topic* |  | *coherence* | *prevalence* | *top_terms_phi* |
| --- | --- | --- | --- | --- |
| t_1 |  | 0.06 | 4.95 | data, research, purpose, data_use, data_would, specific, how_data, anonymise, would_use, study |
| t_2 |  | 0.06 | 5.06 | dont_see, relevant, relevance, not_sure, link, see_relevance, cant_see, see_why, nothing, relevant_health |
| t_3 |  | 0.02 | 3.95 | information, give, insurance, health, wrong, information_could, company, health_insurance, affect, provide |
| t_4 |  | 0.10 | 4.05 | shop, health, habit, interest, shop_habit, wouldnt_want, issue, healthcare, people, business |
| t_5 |  | 0.13 | 5.74 | benefit, understand, would_need, why_would, dont_understand, link, understand_why, not_sure, necessary, unsure |
| t_6 |  | 0.10 | 7.08 | information, more_information, would_use, information_would, would_need, would_want, need_know, information_use, know_why, want_know |
| t_7 |  | 0.08 | 3.59 | make, purchase, lifestyle, relate, food, assumption, decision, alcohol, base, patient |
| t_8 |  | 0.17 | 5.84 | share, information, personal, information_share, personal_information, dont_want, share_information, nothing, data, data_share |
| t_9 |  | 0.09 | 3.66 | medical, information, hold, record, medical_record, doctor, people, supermarket, medical_information, no_need |
| t_10 |  | 0.47 | 6.78 | change, mind, change_mind, nothing, would_change, nothing_would, dont_think, wouldnt_change, make, nothing_change |
| t_11 |  | 0.04 | 4.54 | information, private, personal, access, company, sell, nhs, health, personal_information, party |
| t_12 |  | 0.07 | 4.34 | stored, safe, secure, information, not_sure, 100, 100_percent, percent, convince, information_stored |
| t_13 |  | 0.18 | 4.19 | feel, privacy, big_brother, brother, invasion, invasion_privacy, regard, nothing, dont_feel, feel_like |
| t_14 |  | 0.10 | 5.98 | data, security, trust, dont_trust, data_security, organisation, hack, system, nhs, worry |
| t_15 |  | 0.34 | 7.17 | card, loyalty, store, loyalty_card, store_card, store_loyalty, health, card_health, link, dont_use |
| t_16 |  | 0.13 | 4.88 | buy, shop, family, eat, people, food, supermarket, diet, data, product |
| t_17 |  | 0.08 | 4.29 | concern, privacy, concern_about, confidentiality, detail, data, research, access, team, would_concern |
| t_18 |  | 0.14 | 4.17 | reason, data, data_protection, protection, reason_why, link, good, cant_see, connection, see_reason |
| t_19 |  | 0.26 | 5.93 | health, record, health_record, link, link_health, nothing, private, why_would, need_link, would_link |
| t_20 |  | 0.15 | 3.81 | depend, idea, dont_like, like_idea, would_depend, depend_information, research, incentive, contact, potentially |

Topic model 3

| *topic* | *coherence* | *prevalence* | *top_terms_phi* |
| --- | --- | --- | --- |
| t_1 | 0.26 | 5.93 | health, record, health_record, link, link_health, nothing, private, wouldnt_want, confidential, nothing_health |
| t_2 | 0.09 | 3.93 | give, information, idea, dont_like, good, like_idea, wrong, information_could, free, full |
| t_3 | 0.04 | 4.37 | information, private, hold, access, company, feel, detail, worry, sell, dont_feel |
| t_4 | 0.04 | 3.66 | insurance, health, interest, thing, healthcare, life, commercial, company, not_interest, health_insurance |
| t_5 | 0.13 | 5.91 | data, security, trust, data_protection, protection, dont_trust, secure, data_security, hack, system |
| t_6 | 0.47 | 6.81 | change, mind, change_mind, nothing, would_change, nothing_would, dont_think, wouldnt_change, make, nothing_change |
| t_7 | 0.07 | 4.34 | data, concern, privacy, share, concern_about, data_would, data_use, how_data, data_share, would_concern |
| t_8 | 0.07 | 4.31 | research, depend, data, purpose, specific, would_depend, study, anonymise, happy, collect |
| t_9 | 0.07 | 4.19 | stored, information, secure, safe, 100, 100_percent, percent, not_sure, convince, information_stored |
| t_10 | 0.08 | 4.43 | not_sure, relevant, shop, health, habit, relevance, shop_habit, relevant_health, not_relevant, sure_how |
| t_11 | 0.34 | 7.50 | card, store, loyalty, loyalty_card, store_card, store_loyalty, health, link, card_health, dont_use |
| t_12 | 0.06 | 5.50 | reason, dont_see, link, medical, cant_see, relevance, reason_why, see_relevance, would_need, see_why |
| t_13 | 0.06 | 3.72 | make, purchase, people, good, lifestyle, shop, food, habit, assumption, relate |
| t_14 | 0.12 | 4.85 | buy, shop, family, eat, supermarket, food, people, diet, product, item |
| t_15 | 0.08 | 4.10 | personal, information, benefit, personal_information, health, personal_data, access, personal_health, information_not, evidence |
| t_16 | 0.10 | 5.60 | understand, benefit, would_need, why_would, link, dont_understand, not_sure, understand_why, necessary, unsure |
| t_17 | 0.08 | 7.57 | information, more_information, would_use, information_would, would_want, would_need, information_use, need_know, know_why, want_know |
| t_18 | 0.04 | 4.14 | data, guarantee, research, breach, team, commercial, nhs, access, prefer, safe |
| t_19 | 0.10 | 5.25 | share, information, information_share, dont_want, share_information, nothing, personal, personal_information, nothing_dont, detail |
| t_20 | 0.16 | 3.90 | feel, privacy, big_brother, brother, nothing, invasion, invasion_privacy, feel_like, state, regard |

Topic model 4

| *topic* | *coherence* | *prevalence* | *top_terms_phi* |
| --- | --- | --- | --- |
| t_1 | 0.07 | 4.86 | research, depend, data, purpose, specific, would_depend, team, study, anonymise, happy |
| t_2 | 0.06 | 4.63 | information, private, access, personal, health, data, company, sell, party, nhs |
| t_3 | 0.13 | 4.55 | privacy, data, concern, data_protection, protection, concern_about, invasion, invasion_privacy, confidentiality, would_concern |
| t_4 | 0.06 | 4.00 | data, share, data_would, benefit, purpose, data_share, data_use, how_data, would_use, understand |
| t_5 | 0.05 | 4.29 | information, not_sure, stored, hold, information_stored, medical, safely, safe, people, medical_information |
| t_6 | 0.15 | 5.04 | security, data, secure, 100, 100_percent, data_security, percent, assurance, safe, breach |
| t_7 | 0.13 | 4.49 | data, trust, dont_trust, nhs, organisation, system, commercial, hack, guarantee, security |
| t_8 | 0.14 | 5.67 | information, share, personal, information_share, personal_information, dont_want, share_information, detail, nothing, worry |
| t_9 | 0.13 | 5.71 | understand, benefit, would_need, why_would, dont_understand, understand_why, link, necessary, unsure, benefit_would |
| t_10 | 0.08 | 4.99 | reason, relevance, dont_see, link, cant_see, reason_why, see_relevance, nothing, see_why, should_link |
| t_11 | 0.21 | 4.39 | card, loyalty, loyalty_card, dont_use, interest, use_loyalty, not_interest, dont_know, point, shop |
| t_12 | 0.03 | 3.44 | give, good, convince, if_could, free, full, hand, wrong, permission, would_give |
| t_13 | 0.07 | 3.75 | information, idea, insurance, health, dont_like, like_idea, information_could, company, health_insurance, affect |
| t_14 | 0.20 | 4.17 | feel, make, big_brother, brother, choice, thing, dont_feel, lifestyle, feel_like, state |
| t_15 | 0.12 | 5.48 | buy, shop, family, purchase, food, eat, people, make, diet, relate |
| t_16 | 0.23 | 6.02 | record, health, health_record, relevant, link, not_sure, link_health, medical, medical_record, relevant_health |
| t_17 | 0.47 | 6.87 | change, mind, change_mind, nothing, would_change, nothing_would, dont_think, make, wouldnt_change, think_would |
| t_18 | 0.26 | 6.22 | store, card, health, loyalty, store_card, loyalty_card, link, record, store_loyalty, health_record |
| t_19 | 0.08 | 4.25 | shop, health, nothing, habit, link, shop_habit, wouldnt_want, supermarket, people, regard |
| t_20 | 0.10 | 7.17 | information, more_information, would_use, information_would, would_need, would_want, information_use, know_why, want_know, information_about |

Topic model 5

| *topic* | *coherence* | *prevalence* | *top_terms_phi* |
| --- | --- | --- | --- |
| t_1 | 0.12 | 5.28 | buy, shop, family, food, supermarket, purchase, eat, people, diet, relate |
| t_2 | 0.10 | 7.50 | information, more_information, would_use, information_would, would_need, would_want, information_use, need_know, know_why, want_know |
| t_3 | 0.21 | 3.49 | feel, 100, 100_percent, big_brother, brother, percent, secure, feel_like, thing, watch |
| t_4 | 0.10 | 5.98 | information, share, information_share, personal, dont_want, share_information, personal_information, feel, nothing, want_information |
| t_5 | 0.09 | 5.74 | data, security, trust, dont_trust, guarantee, hack, data_security, nhs, system, organisation |
| t_6 | 0.34 | 7.41 | card, loyalty, store, loyalty_card, store_card, store_loyalty, health, link, card_health, dont_use |
| t_7 | 0.08 | 4.31 | reason, link, reason_why, dont_see, good, would_need, cant_see, explanation, should_link, connection |
| t_8 | 0.20 | 6.07 | health, record, link, health_record, not_sure, relevant, link_health, shop, habit, sure_why |
| t_9 | 0.07 | 4.42 | information, personal, not_sure, stored, personal_information, information_stored, safe, safely, benefit, personal_health |
| t_10 | 0.09 | 3.74 | medical, information, idea, dont_like, record, like_idea, medical_record, doctor, medical_information, give |
| t_11 | 0.06 | 4.48 | information, private, health, company, access, insurance, sell, people, hold, information_about |
| t_12 | 0.13 | 5.30 | understand, benefit, would_need, why_would, dont_understand, understand_why, necessary, link, benefit_would, purpose |
| t_13 | 0.16 | 5.11 | health, record, health_record, relevance, nothing, see_relevance, dont_see, cant_see, private, point |
| t_14 | 0.14 | 4.21 | privacy, interest, invasion, concern, invasion_privacy, confidentiality, not_interest, market, nothing, call |
| t_15 | 0.47 | 6.80 | change, mind, change_mind, nothing, would_change, nothing_would, dont_think, wouldnt_change, make, nothing_change |
| t_16 | 0.10 | 4.56 | research, depend, data, purpose, would_depend, team, access, anonymise, happy, research_team |
| t_17 | 0.08 | 3.55 | make, people, good, lifestyle, shop, regard, purchase, lot, habit, thing |
| t_18 | 0.06 | 3.47 | security, detail, worry, information, worry_about, nanny, improve, party, nanny_state, state |
| t_19 | 0.08 | 3.72 | give, convince, specific, permission, research, benefit, study, data, incentive, if_could |
| t_20 | 0.14 | 4.86 | data, concern, share, data_protection, protection, data_use, data_would, data_share, concern_about, how_data |

Health and fitness app question

Thematic colour key:

|  | Same answer as question 4 |
| --- | --- |
|  | Big brother and data privacy |
|  | Data accuracy |
|  | Don’t use services |
|  | Understanding purpose and process |
|  | Nothing would change mind |
|  | Personal information sharing |
|  | Who has access to the data |
|  | Data security and protection |

Topic Model 1

| *topic* | *coherence* | *prevalence* | *top_terms_phi* |
| --- | --- | --- | --- |
| t_1 | 0.18 | 6.02 | health, record, health_record, link, access, researcher, people, health_researcher, dont_want, lifestyle |
| t_2 | 0.05 | 6.97 | information, would_need, more_information, would_use, detail, benefit, need_more, information_would, would_want, understand |
| t_3 | 0.46 | 6.29 | change, mind, change_mind, nothing, would_change, dont_think, nothing_would, make, think_anything, wouldnt_change |
| t_4 | 0.06 | 4.87 | benefit, health, dont_know, care, would_benefit, how_would, professional, health_care, health_professional, benefit_health |
| t_5 | 0.16 | 3.57 | q4, answer, answer_q4, see_answer, response, response_q4, about_health, access_data, action, affect |
| t_6 | 0.08 | 5.08 | personal, information, private, access, personal_information, dont_want, personal_use, private_information, information_personal, access_information |
| t_7 | 0.14 | 4.36 | feel, privacy, life, big_brother, brother, good, feel_like, watch, invasion, healthy |
| t_8 | 0.03 | 5.78 | trust, secure, nothing, safe, 100, 100_percent, percent, dont_trust, hack, computer |
| t_9 | 0.05 | 5.22 | data, share, data_would, protection, data_could, data_not, data_protection, if_data, share_data, breach |
| t_10 | 0.06 | 5.27 | information, share, information_share, information_wouldnt, private, share_information, wouldnt_share, know_information, internet, store |
| t_11 | 0.07 | 4.44 | not_sure, secure, sure_how, sure_would, would_secure, sure_about, worry, secure_would, worry_about, would_use |
| t_12 | 0.06 | 4.62 | depend, would_depend, information, reason, purpose, give, depend_how, depend_information, research, depend_use |
| t_13 | 0.08 | 4.83 | research, specific, happy, permission, study, purpose, information, condition, time, only_if |
| t_14 | 0.07 | 4.08 | idea, phone, step, good, exercise, dont_like, hold, like_idea, give, count |
| t_15 | 0.03 | 5.64 | security, data, concern, assurance, privacy, safety, data_security, concern_about, internet, about_security |
| t_16 | 0.13 | 4.86 | company, insurance, store, make, lifestyle, interest, nhs, securely, insurance_company, medical |
| t_17 | 0.09 | 3.85 | relevant, point, dont_see, fitbit, wear, not_relevant, see_need, see_point, see_how, provide |
| t_18 | 0.25 | 4.18 | 4, question, answer, question_4, previous, buy, shop, previous_answer, see_previous, affect |
| t_19 | 0.11 | 4.84 | app, accurate, fitness, use_app, not_accurate, activity, dont_think, fitness_app, user, dont_use |
| t_20 | 0.12 | 5.22 | device, use_device, dont_use, data, app, not_use, wearable, collect, wear, future |

Topic Model 2

| *topic* | *coherence* | *prevalence* | *top_terms_phi* |
| --- | --- | --- | --- |
| t_1 | 0.03 | 5.73 | data, security, share, data_would, data_security, protection, data_protection, if_data, share_data, breach |
| t_2 | 0.14 | 4.65 | company, health, insurance, lifestyle, make, nhs, insurance_company, interest, medical, monitor |
| t_3 | 0.20 | 5.12 | record, health, health_record, link, access, relevant, access_health, doctor, not_relevant, not_want |
| t_4 | 0.13 | 5.23 | device, accurate, use_device, app, not_accurate, dont_use, not_use, wearable, device_not, wearable_device |
| t_5 | 0.05 | 4.84 | benefit, health, dont_know, how_would, care, would_benefit, health_care, link, benefit_health, treatment |
| t_6 | 0.46 | 6.24 | change, mind, change_mind, nothing, would_change, dont_think, nothing_would, make, think_anything, wouldnt_change |
| t_7 | 0.14 | 4.44 | access, people, understand, concern, information, good, buy, concern_about, store_card, food |
| t_8 | 0.07 | 4.44 | not_sure, secure, sure_how, sure_would, would_secure, sure_about, secure_would, illegible, situation, if_would |
| t_9 | 0.29 | 4.23 | q4, 100, 100_percent, percent, answer, answer_q4, see_answer, response, guarantee, convince |
| t_10 | 0.07 | 6.44 | information, share, personal, private, information_share, dont_want, personal_information, information_wouldnt, personal_use, private_information |
| t_11 | 0.07 | 4.60 | health, data, researcher, professional, individual, health_professional, health_researcher, want_know, would_want, link |
| t_12 | 0.12 | 4.29 | app, fitness, use_app, dont_use, fitness_app, phone, lifestyle, app_not, user, applicable |
| t_13 | 0.10 | 4.22 | wear, phone, step, hold, exercise, run, device, count, walk, wear_device |
| t_14 | 0.24 | 4.14 | 4, answer, question, previous, question_4, dont_see, point, previous_answer, see_previous, affect |
| t_15 | 0.01 | 5.23 | security, information, store, privacy, internet, concern, assurance, securely, safety, store_securely |
| t_16 | 0.06 | 5.34 | trust, secure, nothing, safe, dont_trust, computer, hack, internet, not_safe, fully |
| t_17 | 0.14 | 4.56 | feel, idea, privacy, big_brother, brother, feel_like, dont_like, life, watch, good |
| t_18 | 0.10 | 4.75 | research, depend, purpose, happy, would_depend, study, depend_why, research_purpose, use_research, depend_how |
| t_19 | 0.05 | 6.55 | information, would_need, more_information, detail, would_use, need_more, information_would, need_know, benefit, information_use |
| t_20 | 0.02 | 4.96 | information, depend, research, give, specific, only_if, permission, if_information, time, reason |

Topic Model 3

| *topic* | *coherence* | *prevalence* | *top_terms_phi* |
| --- | --- | --- | --- |
| t_1 | 0.10 | 5.35 | depend, would_depend, research, specific, study, give, permission, information, purpose, depend_how |
| t_2 | 0.06 | 4.83 | benefit, health, dont_know, care, would_benefit, point, dont_see, how_would, health_care, treatment |
| t_3 | 0.18 | 6.11 | health, record, health_record, access, link, researcher, professional, information, individual, issue |
| t_4 | 0.06 | 5.49 | data, concern, security, share, concern_about, protection, data_not, data_protection, data_security, system |
| t_5 | 0.09 | 4.81 | data, research, want_know, happy, would_want, purpose, personal, data_use, confident, collect |
| t_6 | 0.14 | 4.74 | device, use_device, dont_use, app, wearable, not_use, wearable_device, future, device_not, device_app |
| t_7 | 0.16 | 3.56 | q4, answer, answer_q4, see_answer, response, response_q4, about_health, access_data, action, affect |
| t_8 | 0.04 | 4.59 | information, private, store, personal, personal_use, securely, private_information, store_securely, information_personal, information_store |
| t_9 | 0.35 | 4.88 | security, 100, 100_percent, percent, assurance, internet, information, guarantee, about_security, safety |
| t_10 | 0.07 | 4.37 | not_sure, secure, sure_how, sure_would, would_secure, sure_about, convince, secure_would, illegible, situation |
| t_11 | 0.08 | 5.17 | trust, secure, nothing, dont_trust, computer, hack, worry, website, not_trust, nothing_dont |
| t_12 | 0.06 | 6.85 | information, would_need, more_information, would_use, benefit, understand, need_more, information_would, detail, need_know |
| t_13 | 0.03 | 4.10 | safe, access, people, buy, free, food, not_safe, store_card, weight, unsure |
| t_14 | 0.11 | 5.05 | app, accurate, fitness, use_app, not_accurate, activity, phone, dont_think, fitness_app, reliable |
| t_15 | 0.08 | 5.75 | information, share, personal, information_share, dont_want, personal_information, information_wouldnt, know_information, share_information, wouldnt_share |
| t_16 | 0.14 | 3.92 | wear, relevant, fitbit, device, not_relevant, wear_device, cant_see, fit, easy, time |
| t_17 | 0.46 | 6.31 | change, mind, change_mind, nothing, would_change, dont_think, nothing_would, make, think_anything, wouldnt_change |
| t_18 | 0.14 | 4.97 | company, health, insurance, lifestyle, make, interest, research, information, nhs, insurance_company |
| t_19 | 0.04 | 4.84 | feel, good, idea, step, life, big_brother, brother, exercise, feel_like, dont_like |
| t_20 | 0.11 | 4.31 | privacy, 4, answer, question, previous, question_4, invasion, invasion_privacy, dont_know, previous_answer |

Topic Model 4

| *topic* | *coherence* | *prevalence* | *top_terms_phi* |
| --- | --- | --- | --- |
| t_1 | 0.09 | 3.87 | information, information_would, would_use, secure, worry, reason, store, hack, safe, make |
| t_2 | 0.04 | 4.16 | phone, step, relevant, safe, hold, not_relevant, work, fitbit, exercise, wear |
| t_3 | 0.07 | 4.32 | not_sure, secure, sure_how, sure_would, would_secure, secure_would, sure_about, illegible, situation, how_secure |
| t_4 | 0.31 | 5.30 | information, share, 100, 100_percent, percent, information_share, information_wouldnt, guarantee, share_information, wouldnt_share |
| t_5 | 0.15 | 6.79 | record, information, health, personal, health_record, private, access, share, dont_want, personal_information |
| t_6 | 0.16 | 3.63 | q4, answer, answer_q4, see_answer, response, response_q4, cant_imagine, imagine, about_health, access_data |
| t_7 | 0.06 | 4.90 | benefit, health, dont_see, point, care, would_benefit, condition, link, health_care, how_would |
| t_8 | 0.05 | 5.39 | data, would_need, benefit, understand, need_know, would_use, dont_know, data_would, convince, know_more |
| t_9 | 0.10 | 5.55 | health, company, lifestyle, insurance, researcher, interest, professional, research, data, health_professional |
| t_10 | 0.07 | 4.96 | security, data, assurance, data_security, about_security, worry, would_want, matter, security_information, want_know |
| t_11 | 0.11 | 4.24 | 4, privacy, answer, question, previous, question_4, invasion, invasion_privacy, previous_answer, see_previous |
| t_12 | 0.11 | 6.04 | app, device, accurate, use_device, dont_use, fitness, use_app, not_accurate, wearable, not_use |
| t_13 | 0.12 | 4.38 | concern, people, access, security, concern_about, free, buy, food, control, store_card |
| t_14 | 0.04 | 4.04 | device, store, securely, wear, privacy, store_securely, person, internet, wear_device, intrusive |
| t_15 | 0.04 | 5.06 | data, share, protection, nhs, data_could, data_not, data_protection, share_data, breach, patient |
| t_16 | 0.08 | 4.97 | trust, nothing, secure, dont_trust, computer, internet, fully, safe, not_trust, nothing_dont |
| t_17 | 0.15 | 4.36 | feel, idea, big_brother, brother, good, feel_like, make, dont_like, watch, life |
| t_18 | 0.07 | 5.84 | information, more_information, would_need, detail, information_use, need_more, would_want, if_information, why_would, anonymous |
| t_19 | 0.46 | 6.22 | change, mind, change_mind, nothing, would_change, dont_think, nothing_would, think_anything, make, wouldnt_change |
| t_20 | 0.12 | 5.99 | depend, research, would_depend, study, purpose, give, permission, happy, specific, if_use |

Topic Model 5

| *topic* | *coherence* | *prevalence* | *top_terms_phi* |
| --- | --- | --- | --- |
| t_1 | 0.06 | 4.60 | feel, good, idea, life, big_brother, brother, feel_like, dont_like, watch, make |
| t_2 | 0.23 | 4.42 | q4, answer, 4, question, question_4, previous, answer_q4, see_answer, previous_answer, see_previous |
| t_3 | 0.46 | 6.22 | change, mind, change_mind, nothing, would_change, dont_think, nothing_would, think_anything, make, wouldnt_change |
| t_4 | 0.04 | 4.47 | not_sure, secure, privacy, sure_how, sure_would, would_secure, sure_about, secure_would, illegible, situation |
| t_5 | 0.05 | 4.86 | research, specific, permission, happy, free, relevant, study, give, not_relevant, time |
| t_6 | 0.11 | 4.65 | health, company, insurance, make, interest, lifestyle, research, insurance_company, medical, nhs |
| t_7 | 0.19 | 6.24 | health, record, health_record, access, information, researcher, link, professional, dont_want, health_professional |
| t_8 | 0.06 | 4.67 | depend, would_depend, information, purpose, give, research, depend_how, depend_information, depend_use, depend_why |
| t_9 | 0.07 | 4.77 | benefit, health, point, care, would_benefit, dont_see, condition, dont_know, link, information |
| t_10 | 0.05 | 4.87 | data, benefit, would_need, understand, would_use, convince, data_would, q4, clear, how_would |
| t_11 | 0.10 | 4.73 | accurate, wear, phone, step, not_accurate, device, activity, fitbit, hold, give |
| t_12 | 0.12 | 4.77 | device, use_device, dont_use, app, data, collect, not_use, wearable, wearable_device, future |
| t_13 | 0.08 | 5.02 | trust, nothing, secure, dont_trust, computer, hack, internet, fully, website, not_trust |
| t_14 | 0.09 | 4.85 | data, share, data_not, not_share, if_data, share_data, breach, data_could, nhs, sure_data |
| t_15 | 0.07 | 5.94 | information, share, personal, private, information_share, dont_want, personal_information, personal_use, private_information, share_information |
| t_16 | 0.03 | 5.92 | security, data, concern, privacy, assurance, internet, data_security, protection, concern_about, safety |
| t_17 | 0.12 | 4.26 | app, fitness, use_app, dont_use, fitness_app, app_not, user, phone, lifestyle, applicable |
| t_18 | 0.35 | 4.53 | information, 100, 100_percent, percent, secure, worry, guarantee, information_would, party, information_wouldnt |
| t_19 | 0.07 | 4.31 | store, safe, information, securely, people, not_safe, store_securely, information_store, necessary, buy |
| t_20 | 0.11 | 5.89 | information, would_need, more_information, detail, need_more, would_want, information_use, need_know, know_more, why_would |
