# Supplementary material for: Understanding Barriers to Novel Data Linkages: Topic Modeling of the Results of the LifeInfo Survey
Source: J Med Internet Res. 2021 May 17;23(5):e24236. doi: 10.2196/24236 (PMC8167605; doi:10.2196/24236)
Supplement: Multimedia Appendix 9 [file jmir_v23i5e24236_app9.pdf]

NHS
The Leeds Teaching Hospitals
NHS Trust

What do you think about researchers using your lifestyle information?

We want you to tell us what you think about giving researchers access to information about you and your lifestyle to help improve the health of the community.

What is the purpose of this study?

We don't want you to give us any information about your lifestyle now. What we do want are your thoughts about allowing health researchers to access this information <u>in the future</u>.

What do I have to do?

We would like you to fill in a short survey. It will only take a couple of minutes of your time. You only need to complete this questionnaire once.

Background information

Most people are aware that a good diet and exercise are important for our health. Research into diet exercise and health often involves people filling out long surveys or keeping diaries about what they have eaten and drunk and how much exercise they take. This can take a long time and people find it difficult to remember.

A group of doctors, nurses and researchers at The Leeds Teaching Hospitals NHS Trust and the University of Leeds believe there may be an easier way to carry out this type of research by using information that is already collected electronically and linking it with your health record. This information could be from supermarket loyalty cards such as: Sainsbury's Nectar Card and Tesco Clubcard, and from diet or fitness websites, wearable devices or smartphone apps such as: myfitnesspal, fitbit and shealth/applehealth. Information in your health record from your GP or hospital doctor would include things like whether you have ever had diabetes, heart problems or cancer. We could then study whether people with conditions like these have a different type of lifestyle to those who do not. Lifestyle and health record information once combined would be anonymous and you would never be identified. This sort of research could only be done with your permission.

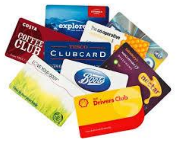

Loyalty card information will include everything you buy in the supermarket from food and drink to health and beauty, home and garden and pet supplies. Information from websites, wearable devices or smartphone apps may include any diet or physical activity information you have chosen to record, along with the location information recorded by your phone, if you have activated this. Examples include the number of steps recorded on your fitbit or what you ate recorded on myfitnesspal. You can read more about examples of these technologies on the NHS website http://tinyurl.com/nhs-lifestyle.

If these links were to be made, in a future study, all information would be stored on secure computers. It would only be accessed by the researchers. No information concerning your individual lifestyle habits would be shared with your Doctor; nor would any health information be shared with any entity outside the research team.

In the current study we only want to find out if you would be prepared to share this information with researchers in the future. Your answers do not commit you to anything. We are interested in your views on this subject. We will not be able to access information about you as a result of anything you say in this survey.

Do I have to take part?

No, taking part is entirely voluntary. Whether you take part or not will have no impact on the care that you receive. By completing the survey you give your consent for researchers to use the information you provide.

What are the benefits of taking part?

There are no direct benefits of participating in this study. The information that you give may help researchers to better understand opinions of linking consumer and health data. In future studies linking this information may help to improve the health of patients

What will I have to do?

If you decide to take part all you have to do is fill in the questions over the page and then hand it in at reception. Alternatively, you can fill in the questionnaire on line by visiting:
https://leeds.onlinesurveys.ac.uk/lifeinfo-ltht

Will my information be kept confidential?

Your personal information (name or address) will not be needed for this study as you will complete the questionnaire anonymously. The questionnaire data will be stored securely at the University of Leeds and will be destroyed 10 years after the end of the study.

Your post code will be used to understand what type of neighbourhood you live in and whether different groups of people respond differently to the questions. It will not be used directly in the research or to identify you.

What will happen to the results of this study?

The results will tell us whether the public are willing in principle for research involving linkage of lifestyle and health information to go ahead in the future. It is likely that general results will be published in an academic journal. We may include direct quotes from your answers, but these will be anonymous and any potentially identifiable information such as names or places will be destroyed. All results will be made available on our study webpage http://lida.leeds.ac.uk/research/lifeinfosurvey/ for you to read.

Who is organising the research?

Doctors, nurses and researchers working at Leeds Teaching Hospital NHS Trust and University of Leeds are jointly organising the study.

The Chief Investigators and those responsible for this study are Dr H Iles-Smith, Head of Nursing Research and Innovation at Leeds Teaching Hospitals, telephone 0113 206 606836, or email heather.iles-smith@nhs.net , Dr M Morris, University Academic Fellow in Health Data Analytics, Leeds Institute for Data Analytics, School of

Medicine, University of Leeds, M.Morris@leeds.ac.uk and Professor A Glaser, Professor of Paediatric Oncology and Late Effects, University of Leeds, a.glaser@nhs.net .

The study is sponsored by Leeds Teaching Hospitals NHS Trust.

Who has reviewed the study?

All research in the NHS is looked at by an independent group of people, known as a Research Ethics Committee to protect your safety, rights, wellbeing and dignity.

This study has been proportionately reviewed by the London-Brent Research Ethics Committee.

What if something goes wrong?

Given that this study is a simple questionnaire, we do not expect anyone to be harmed by this study. However, if you have any complaints about how this study was conducted then please contact either:

Dr H Iles-Smith, Head of Nursing Research and Innovation, Corporate Nursing, Research and Innovation Centre, St James's University Hospital, Beckett Street, Leeds LS9 7TF. E: heather.iles-smith@nhs.net

T: 0113 206 0455, or

Leeds Teaching Hospitals NHS Trust Patient Advice and Liaison Service (PALS) and Complaints http://www.leedsth.nhs.uk/patients-visitors/patient-and-visitor-information/patient-advice-and-liaison-service-pals-and-complaints/ E: patientexperience.leedsth@nhs.net T: (0113) 2066261 (9.00am to 4.30pm Mon to Fri).

This study is covered by NHS Indemnity Scheme. In the event that something does go wrong and you are harmed during the research and this is due to someone's negligence then you may have grounds for a legal action for compensation against Leeds Teaching Hospitals NHS Trust but you may have to pay your legal costs. The normal National Health Service complaints mechanism will still be available to you (if appropriate).

Contact details

You are very welcome to contact us with any questions.

Email the team: leedsth-tr.newdata@nhs.net

Telephone the team: 0113 206 0484 or 0113 206 0479

Sister Linda Bamford, Lead Nurse for Research
Kinga Dwornik, Data Officer

Thank you for your time.
